# Exposure assessment of benzene, toluene, and xylene in petrol station attendants: methods, biomarkers, and the case for integrated monitoring: a narrative review

**DOI:** 10.3389/fpubh.2026.1920988

**Published:** 2026-10-14

**Authors:** Mary O. Balogun, Christopher O. Olopade, Folashade O. Omokhodion

**Affiliations:** 1 Department of Community Medicine, College of Medicine, University of Ibadan, Ibadan, Nigeria; 2 Department of Occupational Health and Safety, University College Hospital, Ibadan, Nigeria; 3 Section of Pulmonary, Critical Care and Sleep, Department of Medicine, Biological Sciences Division, University of Chicago, Chicago, IL, United States

**Keywords:** benzene, biological monitoring, exposure assessment, genotoxicity, LMIC, occupational health, petrol station attendants, toluene

## Abstract

**Background:**

Petrol station attendants (PSAs) are chronically exposed to benzene, toluene, and xylene (BTX) — hazardous volatile organic compounds present in commercial petrol — through daily fuel dispensing, yet occupational BTX monitoring data, particularly from Low- and Middle-Income Countries (LMICs), remain critically limited. Comprehensive exposure assessment of BTX in occupational settings spans four complementary monitoring approaches, organised here using the Environmental Public Health Paradigm (EPHP) as a conceptual lens: environmental monitoring, personal monitoring, biological monitoring, and biological-effect monitoring. The extent to which published studies have simultaneously applied all four monitoring domains among PSAs remains unknown.

**Methods:**

A literature search was performed in PubMed, Medline, and Google Scholar for original articles published from January 1992 to December 2024. This narrative review considered studies reporting data on at least one of the four monitoring domains among PSAs or comparable petrol sector workers.

**Results:**

Environmental and personal monitoring document elevated BTX concentrations at refuelling sites — frequently exceeding international occupational exposure limits (OELs) in LMIC settings — with benzene concentrations ranging from 0.1 to over 6 ppm across reviewed studies. Biological monitoring confirms systemic benzene absorption, with urinary S-phenylmercapturic acid (SPMA) and urinary benzene (U-BEN) emerging as the most sensitive and specific biomarkers. Biological-effect monitoring documents consistent genotoxicity, haematotoxicity, oxidative stress, and immunological disruption in PSA populations globally. To our knowledge, no published study identified in this review has simultaneously implemented all four monitoring domains in a single PSA cohort.

**Conclusion:**

BTX exposure among PSAs constitutes a significant and underaddressed occupational health problem, with a disproportionate burden in LMICs. The absence of integrated multi-domain monitoring evidence limits the development of evidence-based occupational health policy for this vulnerable workforce. Future studies combining all four monitoring domains in a single, well-characterised cohort are recommended.

## 1 Introduction

Petrol/automobile refuelling stations belong to the downstream sector of the oil and gas industry. Petrol station attendants (PSAs) — the individuals who dispense Premium Motor Spirit (PMS) and other petrochemical products — can experience chronic, often inadequately monitored exposure to Volatile Organic Compounds (VOCs). Benzene, toluene, and xylene (BTX) — the principal aromatic fractions of commercial petrol — are among the most hazardous VOCs in occupational settings (1). According to the World Health Organization (WHO), benzene is a Group 1 human carcinogen causally linked to acute myeloid leukaemia and other haematological malignancies, with no identified safe threshold for leukaemogenic effects (2, 3). Notably, the International Agency for Research on Cancer (IARC) and the World Health Organization (WHO) have recently classified gasoline itself as a Group 1 carcinogen (IARC Monographs Volume 138, 2026; available at: https://publications.iarc.who.int/652), further strengthening the evidence base for occupational health protection among PSAs globally. Exposure to fuel vapours and BTX has been associated with neurological, respiratory, and genotoxic effects among exposed workers (4–9).

In most LMICs, self-service refuelling is absent; PSAs manually dispense fuel throughout each work shift, typically without respiratory protection, engineering controls, or occupational health surveillance. Nigeria illustrates this LMIC context: occupational health monitoring and protective practices among petrol station attendants remain concerns (10). Soltanpour et al. (11) reported elevated BTEX concentrations at petrol stations in some Iranian cities, while Attaqwa et al. (12) examined occupational benzene exposure among petrol station workers.

Exposure assessment is an essential prerequisite for establishing the need for preventive measures in occupational health. This review uses four monitoring domains adapted from the Environmental Public Health Paradigm (EPHP) as a conceptual organising lens to structure the evidence base: environmental monitoring, personal monitoring, biological monitoring, and biological-effect monitoring. The EPHP was originally developed in the environmental health context to describe the continuum from environmental contamination to human disease (13, 76), and is applied here to the occupational setting because the same exposure continuum — from source, through ambient and personal air, to internal dose and biological response — applies directly to the PSA worksite. This framework facilitates a structured cross-domain comparison that has not been undertaken in previous PSA-focused literature.

## 2 The environmental public health paradigm as an organising lens for BTX exposure assessment

The Environmental Public Health Paradigm (EPHP) is described in the US EPA Report on the Environment (13, 76). It illustrates the broad continuum of factors or events involved in the potential development of human disease following exposure to an environmental contaminant, progressing from: contaminant release from source → transport via air, water, or land → entry into the human body → biological dose → adverse health effect. Although developed for population-level environmental surveillance rather than occupational exposure assessment, the paradigm’s sequential logic maps directly onto the occupational monitoring continuum in petrol station settings. Applied here as a conceptual organising lens for BTX exposure among PSAs, the four domains are:

### 2.1 Environmental monitoring

Measurement of BTX concentrations in ambient air at or near fuel pumps and in the general worksite environment. Environmental monitoring establishes the worksite hazard but does not capture individual absorbed dose or non-inhalation absorption routes.

### 2.2 Personal monitoring

Measurement of BTX concentrations in the individual worker’s breathing zone, using personal lapel or badge samplers worn during a defined sampling period. Personal monitoring captures individual exposure accounting for work pattern and proximity to source, but does not capture dermal or incidental ingestion routes.

### 2.3 Biological monitoring

Measurement of BTX compounds or their metabolites in blood or urine, reflecting total absorbed internal dose via all routes of exposure. Key urinary biomarkers include urinary benzene (U-BEN), S-phenylmercapturic acid (SPMA), trans, trans-muconic acid (ttMA), hippuric acid (HA), and methylhippuric acid (MHA).

### 2.4 Biological-effect monitoring

Measurement of early adverse biological responses to BTX absorption, including genotoxicity markers, haematological parameters, oxidative stress markers, pulmonary function indices, and immunological alterations. It is acknowledged that biological-effect monitoring, as used here, extends beyond exposure assessment in the classical occupational hygiene sense. In this review, it is included as a downstream component of the exposure-dose-effect continuum to facilitate discussion of the early biological responses potentially associated with BTX exposure, and is not intended to redefine exposure assessment more broadly than it is conventionally understood.

While aims (i)–(iii) below can each be addressed in separate studies, a single study implementing all four domains simultaneously offers an important additional benefit: it can examine empirical cross-domain associations that may help future researchers and regulators in resource-constrained settings select informative monitoring approaches. For example, it can assess relationships between personal air monitoring concentrations and biological-effect endpoints within the same workers. The practical value of integrated four-domain evidence is therefore not to mandate that all future studies measure all four domains, but to inform cost-effective approaches when resources are constrained. The specific aims are to examine: (i) associations between environmental and personal concentrations; (ii) associations between personal monitoring and biological monitoring; and (iii) associations between biological monitoring and biological-effect endpoints. These observational associations can inform risk characterisation but do not, by themselves, establish causality.

Recent reviews have underscored the fragmentation of monitoring evidence in this field. Anigilaje et al. (10) reviewed Nigerian petrol station data and highlighted limited occupational monitoring evidence among Nigerian petrol station attendants. Lima et al. (14) identified thirteen biomonitoring studies and highlighted the lack of a universally accepted gold standard for biological-effect biomarkers. Hoseini et al. (15) demonstrated in a systematic review that biomonitoring is a powerful but underutilised approach in BTEX exposure assessment. This review updates the evidence base across all four monitoring domains and presents the case for integrated BTX exposure assessment among PSAs.

Given that studies in this area vary substantially in exposure assessment methods, biomarkers, analytical techniques, outcome measures, and study populations, a narrative synthesis was considered the most appropriate approach. The objective was to critically evaluate methodological approaches and identify evidence gaps rather than to quantitatively synthesise findings — a task for which a narrative review is well suited.

The central research question framing this narrative review is: To what extent have published studies applied the four complementary monitoring domains (environmental, personal, biological, and biological-effect monitoring) among petrol station attendants, and what are the methodological implications of the current evidence base for future integrated BTX exposure assessment in this vulnerable occupational group, particularly in Low- and Middle-Income Country settings?

## 3 Methods

### 3.1 Search strategy

A literature search was performed using PubMed, Medline, and Google Scholar for articles published from January 1992 to December 2024. The literature search commenced in 1992 because it corresponds to an early published cytogenetic study involving petrol station attendants (16). The search strategy employed Boolean combinations of the following terms: (petrol OR gasoline OR filling station) AND (attendant OR worker) AND (BTX OR benzene OR toluene OR xylene OR BTEX) AND (environmental monitoring OR personal monitoring OR biomonitoring OR biological monitoring OR genotoxicity OR chromosomal aberration OR oxidative stress OR pulmonary function). Benzene, toluene, and xylene were included explicitly in the search terms. Wildcard operators were not used, as initial scoping confirmed that the primary search terms provided adequate retrieval without wildcard expansion.

### 3.2 Study selection approach

As a narrative review, this study does not claim to be an exhaustive retrieval of all relevant literature. Rather, a pragmatic selection approach was used: original articles reporting quantitative data on BTX exposure (environmental, personal, biological, or biological-effect monitoring) among PSAs or comparable petrol sector workers aged 18 years or older, published in English, were considered for inclusion. Studies not assessing any of the four monitoring domains, pure literature reviews without primary data, and studies focused exclusively on non-petrol-sector occupational exposures were not considered for inclusion. No systematic review platform was used, consistent with the narrative review approach. Consequently, it is acknowledged that some relevant studies may not have been identified — a limitation explicitly discussed. Narrative reviews do not aim to be exhaustive, but the specific studies identified by peer reviewers during revision have been incorporated to strengthen the evidence base.

### 3.3 Data extraction and categorisation

The items in the review tables provided a structured format for extracting information from abstracts and full texts, as available, on study objectives, design, country, population characteristics, sex distribution, presence of a control group, monitoring methods and analytical techniques, biomarkers assessed, actual quantitative findings, and key limitations. Extracted data were categorised into the four EPHP monitoring domains. Substantial heterogeneity in study populations, analytical methods, sampling protocols, and reported effect measures precluded meta-analysis; a narrative synthesis was therefore conducted.

Several studies contributed data to more than one monitoring domain and are discussed across the relevant sections. For environmental and personal monitoring, both active sampling (charcoal-sorbent tubes analysed by GC-FID or GC–MS) and passive sampling (Radiello diffusive badges or OVM-type badges) have been used; gas chromatography–mass spectrometry (GC–MS) can offer greater specificity for compound identification than GC-FID, while sensitivity depends on the analytical protocol. For biological monitoring, urinary S-phenylmercapturic acid (SPMA) and urinary benzene (U-BEN) are useful biomarkers for low-level benzene exposure and offer greater specificity than trans,trans-muconic acid (ttMA), which is confounded by dietary sorbic acid.

## 4 Results

The studies reviewed address all four monitoring domains. Most studies were cross-sectional in design. The majority were conducted in Asia (Thailand, India, Iran), Europe (Italy, Spain), and Latin America (Brazil). Only three studies were identified from sub-Saharan Africa, and none simultaneously performed all four monitoring domains in an African PSA cohort. To our knowledge, no published study identified in this review has simultaneously implemented all four EPHP monitoring domains in a single PSA cohort — a notable evidence gap identified in this review.

### 4.1 Environmental monitoring of BTX among PSAs

Table 1 presents a selection of environmental monitoring studies reviewed for this synthesis. Several studies reported higher ambient BTX concentrations at petrol station worksites than at comparison sites, although sampling locations and methods differed. Zoleikha et al. documented operator personal benzene of 1.28 ppm, approximately 2.5 × the Iranian TLV-TWA of 0.5 ppm, while a maximum ambient benzene value of 5.103 ppm (~10 × the 0.5 ppm benchmark) was reported at some sampling points (17). Allahabady et al. (18) reported mean ambient benzene of 2.785 ± 1.461 mg/m^3^; the cited paper states an occupational limit of 0.5 ppm (approximately 1.6 mg/m^3^ after correct conversion), below the reported overall mean. In Thailand, Chaiklieng (19) found that 5 of 47 stations produced a combined BTEX Hazard Index exceeding 1, with 2 of 47 stations (4.3%) exceeding the 100 ppb benzene benchmark used in the study.

**Table 1:** Environmental monitoring of BTX/BTEX exposure at petrol/gasoline station worksites.

Ref	Author, Year Country	Population/sites	Sampling duration and method	Analytical method	Quantitative results	Interpretation
(20)	Gonzalez-Flesca et al., 2002 — France	• 1 motorway service station (rural, Prunay). • 3 proximity levels: near pumps, surrounding environment, background. • 20 sampling locations.	• 2-week campaign (Nov 1999). • Week 1: Stage II vapour recovery ON. • Week 2: Stage II OFF. • Passive PE tubes (Carbotrap B adsorbent); 48-h deployments at Level I; longer at Levels II–III.	Thermal desorption + GC-FID (WCOT column 50 m × 0.32 mm, CP 5/2.5 CB).	• Benzene near pumps — Stage II OFF: 3.6–8.06 μg/m^3^. • Benzene near pumps — Stage II ON: 2.56–3.5 μg/m^3^. • Road traffic (RN10) worst-case contribution at surrounding sites: 4.8 μg/m^3^ benzene.	• Stage II vapour recovery reduced benzene by 29–56% in comparisons adjusted for wind speed. • VRS efficiency inversely proportional to wind speed. • Concentrations at this rural station lower than in urban street canyons. • Road traffic is a measurable co-source of benzene even at rural stations.
(21)	Tunsaringkarn et al., 2012 — Thailand	• 49 workers (38 M, 11F) at 6 gasoline stations in Bangkok. • 4 inner-area stations; 2 outer-area stations.	• 8-h shift (06:00–14:00). • Active sampling; charcoal tubes; 100 mL/min. • 2 samples from personnel working area + 1 roadside per station.	• GC-FID (Varian CP3800, CP 52 wax column 30 m × 0.25 μm × 0.25 mm). • LOD: benzene 0.06 ppm; toluene 0.03 ppm.	• Benzene: 107.68 ± 21.55 ppb (station work areas) vs. 68.38 ± 6.31 ppb (roadside). • Toluene: 226.68 ± 51.10 vs. 147.05 ± 37.41 ppb. • Ethylbenzene: 7.25 ± 5.95 vs. 0.01 ± 0.01 ppb. • Xylene: 11.56 ± 5.51 vs. 4.50 ± 0.00 ppb. • Non-cancer HQ (total BTEX) = 0.617. • Cancer risk from benzene = 1.75 × 10^−4^. • B/T ratio: 0.48 at station; 0.47 at roadside.	• No significant difference between inner and outer Bangkok areas. • Non-cancer HQ below acceptable threshold of 1. • Estimated cancer risk (1.75 × 10^−4^) exceeds the upper end of the commonly cited 10^−6^–10^−4^ risk-management range. • Near-identical B/T ratios at station and roadside are consistent with a contribution from road traffic to ambient benzene.
(17)	Zoleikha et al., 2017 — Iran	• 2 fuelling stations in Ahvaz City. • Operators AND customers sampled. • 30 ambient air samples; 15 personal breathing-zone samples. • Operator working duration: 7 h/day. • Customer exposure duration: 30 min.	• Personal samples — operators: 8-h TWA. • Personal samples — customers: 30-min STEL comparison. • SKC low-flow pumps; NIOSH Method 1,501.	GC-FID.	• Personal benzene — operators: mean 1.28 ppm. • Personal benzene — customers: mean 0.42 ppm. • Maximum ambient benzene at some sampling points: 5.103 ppm. • Highest mean ambient benzene — Station 2 car pumps: 1.69 ± 0.84 ppm; motorcycle pumps: 1.63 ± 1.76 ppm. • Toluene, ethylbenzene, xylenes: all below OELs.	• Operators exceeded Iranian TLV-TWA of 0.5 ppm. • Customers below STEL (2.5 ppm). • Maximum ambient benzene approximately 10 × the 0.5 ppm occupational benchmark at some sampling points; ambient and personal samples are not interchangeable. • Only benzene consistently exceeded OEL — toluene, ethylbenzene and xylenes within limits. • Petrol overflow observed as a potential source of exposure.
(23)	Xia et al., 2017 — China	• 144 workers: 96 petrol fillers (refuelling areas) and 48 cashiers (offices). • 9 filling stations in Nanning, Guangxi. • Primary focus: Mn (MMT additive) co-exposure alongside BTEX.	• Ambient air monitoring at refuelling areas (2 sampling points), a convenience store and an office area per station as described in Methods; the figure depicts 2 refuelling points and 1 office point. • Concurrent with biological sample collection.	• BTEX: GC–MS (Agilent); air collected using a QC-4S sampler. • Mn: NIOSH atomic absorption method.	• BTEX TWA: significantly higher in refuelling areas than in office areas (*p* < 0.05). • Mn TWA — refuelling areas: 6.44–127.34 μg/m^3^; office areas: 3.16–7.22 μg/m^3^ (*p* < 0.05). • SOD: 39.18 ± 6.05 vs. 52.84 ± 3.87 U/mL (*p* < 0.001). • MDA: 1.68 ± 0.52 vs. 1.43 ± 0.64 nmol/L (*p* = 0.021). • Hsp70: 2.77 ± 0.64 vs. 2.32 ± 0.87 ng/mL (*p* = 0.002). • BTEX CEI vs. SOD: *r* = −0.508 (*p* < 0.001). • BTEX CEI vs. MDA: *r* = 0.367 (*p* < 0.001).	• All BTEX concentrations below Chinese national occupational standards (GBZ 2.1–2007) despite being significantly higher in refuelling areas than offices. • The exposed group showed differences in oxidative stress biomarkers and higher plasma Hsp70; this study also assessed manganese co-exposure. • BTEX and Mn co-exposure may have combined effects on oxidative stress — an important consideration given the widespread use of MMT as a fuel additive.
(19)	Chaiklieng, 2021 — Thailand	• 47 gasoline stations in Khon Kaen Province. • Fuelling workers and cashiers; all workers ≥18 years.	• 4-h active sampling during peak service hours. • Charcoal tubes; 0.2 L/min; NIOSH Method 1,501; CS₂ extraction.	• GC-FID (Varian CP3800, CP 52 wax column 30 m × 0.25 μm × 0.25 mm). • Reported LOD: <0.003 ppb for benzene; <0.001 ppm for toluene.	• Benzene: mean 33.1 μg/m^3^ (0.1–437.5); ppb range 0.1–136.9. • Toluene: mean 142.7 μg/m^3^ (30.5–1,529.7); ppb range 8.1–406.0. • Ethylbenzene: mean 14.4 μg/m^3^ (3.4–104.9); ppb range 0.8–24.1. • Xylene: mean 41.3 μg/m^3^ (1.7–458.3); ppb range 0.4–105.5. • HI range: <0.1 to 7.3. • HI correlated with daily gasoline sold ≥3,000 L/day (*p* = 0.02) and number of dispensers ≥12 (*p* = 0.05).	• Benzene exceeded NIOSH standard (100 ppb) in 2/47 stations (4.3%). • 5 stations (10.6%) reached HI > 1 — indicating unacceptable combined BTEX health risk. • Hazardous Area I defined as within 4 m radius of dispenser; Area II as 4–8 m. • High-volume stations (≥3,000 L/day) and those with more dispensers (≥12) pose significantly greater risk to workers.
(22)	Salama et al., 2021 — Saudi Arabia	• 40 fuel-dispensing facilities; Dammam and Al Khobar (20 per city). • Locations included fuel stations and nearby residential and street/traffic sites; numbers sampled by location were not uniform.	• 8-h monitoring period. • Active sampling; SKC battery pump PCXR4; 100 mL/min; activated charcoal cartridges. • The paper also describes day and night fixed-site sampling; timing is not consistently specified.	• MIRAN SapphIRe Portable Ambient Analyzer (infrared — not GC-FID). • LOD: 0.05–0.07 μg/L. • See Interpretation column for analytical caveat.	• Benzene — Dammam: mean 10.30 ppm (SD 2.5; range 0.25–21.63). • Benzene — Al Khobar: mean 11.07 ppm (range 0.83–25.05). • By site type (both cities): residential mean 8.0 ppm; side street 12.2 ppm; direct street 11.5 ppm. • Toluene (TLV 20 ppm) and xylene (TLV 100 ppm): within TLV at all sites. • HQ for benzene: residential 1.76; side street 2.34; direct street 3.45.	• Reported city and site-type mean ambient benzene values exceed the cited 0.5 ppm occupational benchmark; individual worker TWAs were not measured. • Reported benzene HQs for residential, side-street and direct-street areas all exceed 1; HQ is a non-cancer risk metric. • Toluene and xylene within permissible limits. • Analytical caveat: because the MIRAN SapphIRe uses infrared spectrometry, method-specific calibration and potential cross-sensitivity should be considered. Comparisons with compound-specific chromatographic measurements should therefore be made cautiously.
(18)	Allahabady et al., 2022 — Iran	• 13 gas stations in Mashhad city. • 39 samples: 3 per station (morning, noon, night). • City divided into 5 areas; stations coded A–M.	• Active sampling; autumn 2018. • SKC Ltd. DT11 8ST pump; 0.2 L/min; activated carbon sorbent tubes. • ~ 75 min per sample; tubes ~2 m above ground.	• GC-FID (Agilent 7,890); CS₂ desorption; column SGE (25 mm × 0.22 mm capillary). • MDL: 0.12–0.16 μg/m^3^; calibration R^2^ ≥ 0.999.	• Benzene: mean 2.785 ± 1.461 mg/m^3^ (range across stations: 1.024–5.635 mg/m^3^). • Toluene: mean 3.495 ± 1.390 mg/m^3^. • Ethylbenzene: mean 2.091 ± 0.811 mg/m^3^. • Xylene: mean 1.140 ± 0.419 mg/m^3^. • Highest station (Code C, city centre): benzene 5.635 ± 1.875 mg/m^3^; toluene 6.324 ± 1.921 mg/m^3^. • Benzene negatively correlated with humidity (*P* < 0.05).	• Iranian occupational limit cited in the paper for benzene = 0.5 ppm (approximately 1.6 mg/m^3^); the paper incorrectly equates 0.5 ppm with 3.2 mg/m^3^. • Overall ambient benzene mean (2.785 mg/m^3^) is approximately 1.74 × the correctly converted 0.5 ppm limit (1.6 mg/m^3^); the paper’s stated 5.5 × comparison results from inconsistent units. • Toluene, ethylbenzene, xylene: all within permissible limits. • City-centre location with high traffic density associated with highest BTEX concentrations. • Engineering measures to reduce fuel evaporation recommended.
(47)	Costa-Amaral et al., 2019 — Brazil	• 51 filling station workers (Group I) from filling stations located along the same busy highway, Rio de Janeiro. • 35 security guard controls at campus entrances on same highway (Group II). • Both groups included smokers; tobacco and alcohol use were recorded.	• 150-min work-area air sampling. • Active samplers (Anasorb CSC, SKC; PCXR4 pump calibrated for NIOSH Method 1,501). • Air samples collected at workplaces of both groups (30 filling-station and 18 comparison-location samples).	• GC-FID (Focus, Thermo-Finnigan; Carbowax 20 M column 60 m × 0.32 mm × 0.25 μm). • LOQ: 5.60 μg/m^3^ benzene; 6.70 μg/m^3^ toluene.	• Benzene: filling-station work areas 14.85 ± 9.85 μg/m^3^ (median 12.96; *n* = 30); comparison sites ≤LOQ (*n* = 18), *p* = 0.0001. B/T ratios: 0.74 (filling stations) vs. 0.23 (comparison locations). • Toluene: filling-station work areas 20.18 ± 14.30 μg/m^3^ (*n* = 30); comparison locations 24.19 ± 67.80 μg/m^3^ (*n* = 18); median 17.03 vs. 6.70 μg/m^3^.	• B/T ratios differed between work areas, consistent with different source contributions alongside shared traffic background. • Multi-domain study: urinary biomarkers (ttMA, SPMA) and multiple genotoxicity endpoints (MCA, chromosome breaks, comet C-FPG, MN) measured in same subjects. • Study spans environmental monitoring, biological monitoring, and biological-effect monitoring domains. • Environmental air results are based on 150-min work-area samples and do not represent full-shift personal exposure.

BTX/BTEX, benzene, toluene, ethylbenzene, xylene; GC-FID, gas chromatography–flame ionisation detection; LOD, limit of detection; μg/m^3^, micrograms per cubic metre; mg/m^3^, milligrams per cubic metre; ppm, parts per million; OEL, occupational exposure limit; TLV-TWA, Threshold Limit Value–Time Weighted Average (ACGIH: benzene 0.5 ppm, 1,600 μg/m^3^); STEL, Short-Term Exposure Limit; HI, Hazard Index; HQ, Hazard Quotient (exposure ÷ reference concentration); NIOSH, National Institute for Occupational Safety and Health; PSA, petrol station attendant; M, male; F, female; VRS, vapour recovery system; B/T, benzene-to-toluene ratio; CEI, cumulative exposure index; SOD, superoxide dismutase; MDA, malondialdehyde; Hsp70, heat shock protein 70; Mn, manganese; MMT, methylcyclopentadienyl manganese tricarbonyl; TWA, time-weighted average. Salama (22): The MIRAN SapphIRe Portable Ambient Analyzer is an infrared instrument, not GC-FID — its method differs from the GC-based methods used in several other studies; interpret reported benzene values cautiously. Zafar et al. (77) reported identical data from the same 40 stations; confirmed as a duplicate of Salama et al. (22) and removed from the reference list.

Gonzalez-Flesca et al. (20) demonstrated that Stage II vapour recovery reduced benzene concentrations near pumps by 29–56%, in wind-adjusted comparisons, with efficiency inversely proportional to wind speed. Tunsaringkarn et al. found that while BTX concentrations were slightly but consistently higher at gasoline stations than at roadsides, the B/T ratios of 0.48 at stations vs. 0.47 at roadsides were consistent with road traffic contributing to benzene in the Bangkok study area (21). Salama et al. (22) reported high mean ambient benzene concentrations of 10.30 ppm in Dammam and 11.07 ppm in Al Khobar, based on measurements at fuel stations and nearby locations in Saudi Arabia. Xia et al. (23) confirmed that BTEX concentrations in refuelling areas were significantly higher than in offices (*p* < 0.05), alongside differences in oxidative stress biomarkers in a study that also assessed manganese exposure, despite BTEX values remaining below the cited Chinese national occupational standards. A critical analytical caveat applies to Salama et al. (22): the MIRAN SapphIRe Portable Ambient Analyzer is an infrared instrument rather than a GC-based method. The unusually high reported values should be interpreted with caution when compared with studies using different sampling and analytical methods.

### 4.2 Personal monitoring of BTX exposure among PSAs

Table 2 presents a selection of personal monitoring studies reviewed for this synthesis. Personal benzene concentrations in PSAs ranged from a median of 0.11 mg/m^3^ (geometric mean 0.12 mg/m^3^) in Italian filling station attendants (24) to a mean of 1.40 ± 0.80 ppm in Iranian petrol station workers (25)—with the highest values, substantially exceeding the ACGIH TLV-TWA of 0.5 ppm, documented in LMIC settings where vapour recovery systems were absent. Scheepers et al. (26) in Sri Lanka documented a personal benzene median of 609 μg/m^3^, with 28% of measurements exceeding the ACGIH TLV-TWA when adjusted for long working hours, and identified specific high-risk tasks including refilling the main fuel storage tank (peak 239 ppm) and measuring fuel levels in storage tanks (peak 96.4 ppm). Campo et al. in Italy documented a median personal benzene concentration of 59 μg/m^3^ in PSAs versus 4 μg/m^3^ in controls—a greater than 14-fold difference in a regulated European setting (27).

**Table 2:** Personal monitoring of BTX/BTEX exposure among petrol station attendants (PSAs).

Ref	Author, Year Country	Population (M/F)	Sampling duration and method	Analytical method	Quantitative results	Interpretation
(24)	Lagorio et al., 1993 — Italy	• 111 PSAs (sex not stated). • 703 personal samples; Rome. • Dec 1991–Nov 1992. • Mean 6.4 sampling days/worker.	• Full 8-h shift. • Charcoal tubes + personal pumps (ALPHA-2 Du Pont sampler); 200 mL/min; NIOSH method.	Gas chromatography.	• Personal benzene (8-h TWA, mg/m^3^): mean 0.55 (SD 2.46); median 0.11; GM 0.12 (GSD 3.82); range 0.001–28.02. • Personal toluene: mean 0.70; median 0.17; GM 0.18. • Personal xylenes: mean 0.32; median 0.09; GM 0.10. • Toluene and xylenes highly correlated with benzene (*r* = 0.87 and *r* = 0.83). • Significant predictors of benzene: super premium gasoline sold (*p* < 0.001); winter season (*p* < 0.001).	• The distribution was strongly right-skewed; the median (0.11 mg/m^3^) was more representative than the mean (0.55 mg/m^3^). • The multivariable model explained 12.3% of exposure variability. • Gasoline type/sales and winter season were reported determinants of individual exposure.
(48)	Carere et al., 1995 — Italy	• 23 male non-smoking PSAs; Rome. • 24 age-matched non-smoking controls. • Mean employment 22.4 years.	• Full shift; active carbon vials + personal pumps; breathing zone. • Repeated sampling for 1 year; mean 6.5 measurements/subject.	Gas chromatography (NIOSH method).	• Personal benzene (8-h TWA): mean 1.5 mg/m^3^ (SE 0.7); range 0.1–13.1 mg/m^3^. • CA: exposed 1.97% vs. controls 1.46%; borderline significant (*p* = 0.066). • SCE: 4.73 ± 0.15 vs. 4.48 ± 0.11 per cell (NOT significant; *p* = 0.181). • HFC (75th percentile): R*R* = 1.3 (95% CI 1.1–1.5; significant). • High benzene sub-group: CA R*R* = 1.60 (95% CI 1.09–2.36); χ^2^ trend *p* = 0.0183. • Blood lead: exposed 10.7 vs. controls 8.4 μg/dL (*p* = 0.036).	• Some individual benzene measurements exceeded the occupational limit used at the time. • CA and SCE were not significantly elevated overall; CA increased with benzene exposure in the higher-exposure subgroup. • Higher blood lead in attendants was consistent with concurrent exposure during the leaded-petrol era.
(49)	Pitarque et al., 1997 — Spain	• 42 male PSAs at 11 stations, Barcelona. • 45 male controls (university campus).	• 16-h total exposure period. • 3 M OVM-3500 passive badge; near breathing zone; CS₂ desorption.	• GC-FID. • Concentrations calculated using 3 M manufacturer formula.	• Personal BTX (8-h TWA, mg/m^3^): benzene 0.91 (workers) vs. 0.06 (control streets). • Toluene: 1.58 vs. 0.28. Xylenes: 0.89 vs. 0.14. • SCE mean: 7.99 ± 0.27 vs. controls 7.38 ± 0.27 per cell. • HFC (95th percentile): 6.18 ± 1.26% vs. 4.38 ± 1.06% (*p* = 0.02 by χ^2^-test).	• All BTX values below EU OEL (3.25 mg/m^3^) and ACGIH TLV (1.6 mg/m^3^) at time of study. • After statistical modelling for lifestyle factors: occupational exposure NOT independently significant for SCE. • Cigarette smoking was the only significant predictor of SCE (*p* < 0.001) — confirms importance of smoking as confounder.
(50)	Romieu et al., 1999 — Mexico	• 27 service station attendants. • 8 street vendors. • 10 office workers. • Downtown Mexico City, 1996.	• Passive OVM badges (3 M and Pro-Tek/DuPont); mean 6 h worn. • Blood: collected beginning and end of shift.	• Badges: GC-FID. • Blood: purge-and-trap GC/isotope dilution MS (CDC laboratory; LOD 0.030 μg/L benzene).	• Personal badge concentrations (μg/m^3^): service-station attendants—benzene GM 310; median 330 (range 130–770). • Street vendors: benzene GM 77; office workers: benzene GM 44. • Attendants: toluene GM 680; ethylbenzene GM 110; m−/p-xylene GM 360. • Blood benzene—attendants at beginning of shift: GM 0.64; median 0.63 μg/L. • Blood benzene post-shift: GM 0.47; median 0.42 μg/L. • Personal-air benzene versus post-shift blood benzene: *r* = 0.25.	• Attendants had significantly higher personal benzene exposure than street vendors and office workers (*p* < 0.0001). • All reported attendant badge benzene concentrations were below the ACGIH TLV-TWA of 1,600 μg/m^3^. • The weak personal-air–blood correlation should be interpreted in light of chronic background exposure, timing of blood collection and the cross-sectional design.
(30)	Periago and Prado, 2005 — Spain	• Service station PSAs in south-east Spain. • 1995: *n* = 21; 2000: *n* = 28; 2003: *n* = 19. • All male; July sampling only.	• Full shift (6.5–8 h). • 3 M-3500 passive OVM badge worn by individual PSAs; breathing zone.	GC-FID.	• Personal benzene (8-h TWA) by survey year: • 1995: mean 736 μg/m^3^ (SD 346; GM 667; range 272–1,603). • 2000: mean 241 μg/m^3^ (SD 87; GM 226; range 114–452). • 2003: mean 163 μg/m^3^ (SD 132; GM 124; range 36–564). • 20% of attendants in 1995 exceeded the then-ACGIH TLV-TWA of 960 μg/m^3^. • Volume of petrol sold and ambient temperature were independent predictors of personal benzene.	• Personal benzene exposure decreased significantly across the three survey periods. • The reduction followed implementation of EU Directive 98/70/EC, which limited petrol benzene content to a maximum of 1% v/v from January 2000. • The repeated surveys provide longitudinal evidence that fuel-composition controls can reduce attendant exposure.
(51)	Raghavan and Basavaiah, 2005 — India	• 29 gasoline handlers. • 30 office staff controls. • Bangalore; winter 2004; all male.	• Full 8-h shift. • Anasorb CSC sorbent tube (SKC 226–01) + personal pumps; 200 mL/min; OSHA Method No. 12.	• Trace GC (Thermo Finnegan) + MXT-624 column + FID. • Urinary ttMA: HPLC-UV; Q-SAX SPE; C8 column; 259 nm.	• Personal benzene TWA—gasoline handlers: mean 1.10 ± 1.08 mg/m^3^; range 0.19–5.27 mg/m^3^. • Controls: mean 0.070 ± 0.035 mg/m^3^. • 6/29 (20.69%) gasoline handlers exceeded the ACGIH TLV of 1.60 mg/m^3^. • Urinary ttMA—gasoline handlers: mean 563.16 ± 281.81 μg/g Cr (range 163.43–1,196.46). • 15/29 workers exceeded the historical ACGIH BEI of 500 μg/g Cr. • Controls: mean 266.88 ± 110.65 μg/g Cr; none exceeded the BEI. • Personal benzene versus urinary ttMA: *r* = 0.442 (*p* < 0.05).	• Mean personal benzene was below the ACGIH TLV, but 20.69% of individual workers exceeded it. • Approximately half of the workers exceeded the historical ttMA BEI, indicating greater internal benzene exposure than controls. • The moderate air–ttMA correlation supports, but does not by itself prove, occupational uptake.
(25)	Bahrami et al., 2007 — Iran	• 25 petrol station workers. • 60 taxi drivers. • 60 non-exposed rural controls. • Hamadan state; all male.	• 2–4 h (recalculated to 8-h TWA). • SKC charcoal tube + Negretti Automation pump (NR645); 200 mL/min; CS₂ desorption. • Urine: end-of-shift.	• GC-FID (Unicam 4,600; glass column 10% SE30; 1.5 m × 4 mm). • Urinary ttMA: HPLC-UV (Knauer K-2600; 259 nm; APEX ODS II 3 μm column).	• Personal benzene (ppm): Petrol station workers: mean 1.40 ± 0.80 (range 0.2–3.1). • Taxi drivers: mean 0.31 ± 0.22 (range 0.07–0.95). • Urinary ttMA (mg/g Cr): Petrol station workers: mean 2.64 ± 0.47 (range 1.20–3.28). • Taxi drivers: mean 0.31 ± 0.44; Controls: mean 0.17 ± 0.16. • All groups significantly different from controls (*p* < 0.05). • Correlation benzene vs. ttMA: PSWs *r* = 0.65 (*p* < 0.05); drivers *r* = 0.30 (poor).	• PSW mean benzene approximately 3 × ACGIH TLV-TWA of 0.5 ppm — significant exceedance. • ttMA unreliable as biomarker when benzene <0.17 ppm due to dietary sorbic acid confounding. • Note: sampling duration was 2–4 h, recalculated to 8-h TWA.
(52)	Bahrami et al., 2008 — Iran	• 25 petrol station workers. • 45 taxi drivers. • Hamadan; all male; March 2003–March 2004.	• 2–4 h (recalculated to 8-h TWA). • SKC charcoal tube + Negretti Automation pump; 200 mL/min; CS₂ desorption. • Urine: end-of-shift.	• GC-FID (Unicam 4,600; glass column 10% SE30). • Urinary MHA: HPLC-UV (Knauer K-2600; 254 nm; APEX ODS II 3 μm; NIOSH method).	• Personal xylenes (ppm): Petrol station workers: mean 1.05 ± 0.55 (range 0.20–2.55; median 0.96; GM 0.92). • Taxi drivers: mean 0.24 ± 0.26 (range 0.00–1.09). • Urinary total MHA (mg/g Cr): Petrol station workers: mean 65.59 ± 42.76 (range 34.39–203.00; median 46.57; GM 57.27). • Taxi drivers: mean 17.30 ± 24.86; significantly different (*p* < 0.05). • Correlation xylenes vs. MHA (PSWs only): o-xylene R^2^ = 0.77; m + p-xylene R^2^ = 0.69 (both *p* < 0.05).	• PSWs approximately 4–5 × higher xylene exposure than taxi drivers; all below ACGIH TLV (100 ppm). • Strong xylene-MHA correlation in PSWs validates MHA as a reliable xylene biomarker in this occupational group. • Poor correlations in taxi drivers (R^2^ = 0.09–0.38) reflect lower and more variable xylene exposure. • Note: sampling duration was 2–4 h, recalculated to 8-h TWA.
(53)	Keretetse et al., 2008 — South Africa	• 20 petrol attendants from 3 stations at Potchefstroom. • 20 unexposed controls. • Matched for age and smoking.	• Full 8-h shift; Friday (highest sales day). • Sorbent tubes + pump at 0.125 L/min; NIOSH Method 1,501 (modified).	GC–MS at accredited laboratory.	• Benzene: mean 0.65 mg/m^3^ (SD 0.47); range 0.06–1.85. • Toluene: mean 0.73 mg/m^3^ (SD 0.41); range 0.08–1.73. • Xylene: mean 0.31 mg/m^3^ (SD 0.16); range 0.04–0.54. • Total VOCs: mean 46.57 mg/m^3^ (SD 29.53); range 3.02–99.61. • Volume of petrol sold correlated with benzene (*r* = 0.634; *p* = 0.004). • Relative humidity negatively correlated with benzene (*r* = −0.497; *p* = 0.03). • DNA damage (comet, tail intensity): exposed 15.06 ± 9.10 vs. unexposed 6.30 ± 3.37 (*p* < 0.05). • After ANCOVA: adjusted means 9.397 vs. 7.638 (*p* < 0.001).	• Reported BTX concentrations were below the South African OELs used in the study. • DNA damage was higher in attendants despite sub-OEL BTX measurements; the cross-sectional design does not establish causation. • Petrol volume and humidity were associated with measured benzene exposure.
(29)	Pandey et al., 2008 — India	• 100 petrol-pump workers from 15 stations, Lucknow; all male. • 100 matched controls.	• Air: real-time PID monitoring. • Blood: GC–MS headspace (Perkin Elmer AutoSystem XL, PE-624 column).	• Air: ppbRAE VOC monitor (dual PID, 10.6 eV UV source). • Blood BTX: GC–MS headspace; SIM mode.	• Air benzene at petrol pump stations: 100–250 ppb; control sites: 5–10 ppb. • Blood benzene — PPWs: mean 7.94 ppb (*n* = 39); controls: mean 2.82 ppb (*p* < 0.05). • Comet assay: OTM 4.14 ± 1.25 vs. controls 3.29 ± 0.92 (*p* < 0.001); tail DNA% and tail length also significantly higher. • MN frequency (subset): 14.79 ± 3.92% vs. 7.54 ± 3.00% (*p* < 0.001). • PPWs with high blood benzene had significantly higher MN than low-blood-benzene PPWs (*p* < 0.001).	• The study linked air BTX, blood BTX and genotoxicity measurements in the same participants. • Blood benzene was significantly higher in workers, supporting occupational uptake. • Workers with higher blood benzene had higher micronucleus frequencies, demonstrating an exposure–effect association rather than proving causation.
(28)	Fracasso et al., 2010 — Italy	• 28 service station attendants (SSA). • 33 petrochemical operators (PIO). • 21 pump maintenance workers (GPMW). • 51 non-exposed controls; all male.	• Full ~8-h shift. • Personal passive sampler (Radiello®, active carbon cartridge); respiratory zone.	• GC-FID (modified NIOSH method 1,501); LOD 3 μg/m^3^. • Urinary ttMA: HPLC-UV; SAX SPE. • Urinary SPMA: HPLC-MS/MS.	• Personal airborne benzene (μg/m^3^; median, range): SSA: 40.00 (8.00–260.00) vs. controls 5.40 (*p* = 0.0001). • Urinary metabolites (μg/g Cr; SSA vs. controls): SPMA: 5.55 (1.55–15.00) vs. 1.90 (*p* = 0.0001). • ttMA: 117.00 (30.00–418.00) vs. 84.00 (*p* = 0.0258). • Comet TM: SSA 0.37 ± 0.13 vs. controls 0.28 ± 0.08 (*p* = 0.002). • CA (subgroup): NOT significantly different. • Correlation personal benzene vs. comet TM: SSA *r* = 0.509 (*p* = 0.007).	• Personal exposure, urinary biomarkers and genotoxicity were assessed in the same participants. • SPMA was significantly elevated and correlated with personal benzene, supporting its sensitivity at sub-OEL exposures. • Comet-assay tail moment differed between groups, whereas chromosomal aberrations did not.
(54)	Moro et al., 2013 — Brazil	• 43 male GSAs (non-smokers). • 28 male controls. • Rio Grande do Sul. • Mean exposure 9.7 ± 1.1 years.	Personal passive sampler (SKC 575–002®); ~8-h shift; CS₂ desorption.	• GC-FID (PerkinElmer; Innowax column 25 m × 0.2 mm × 0.4 μm). • Urinary ttMA: HPLC-UV after SPE. • Comet assay; buccal cell MN assay. • Oxidative stress markers.	• Airborne BTEX (μg/m^3^; median, IQR): Benzene: 144.2 (58.1–2,207.5) GSA vs. 42.0 (34.1–52.4) controls (*p* < 0.001). • Toluene: 345.3 (213.0–3,945.1) vs. 107.2 (78.4–172.3). • o-Xylene: 182.0 (29.8–627.4) vs. 30.0 (21.6–65.3). • Urinary ttMA: significantly higher in GSA vs. controls (*p* < 0.001). • DNA damage index (comet): significantly higher in GSA. • Buccal cell MN: significantly higher in GSA. • Protein carbonyl and 3-nitrotyrosine: significantly higher; SOD, CAT, GST, GSH: significantly lower.	• Median GSA BTEX concentrations were below ACGIH limits, although the upper benzene range included values above the benzene limit. • Personal air monitoring, urinary biomonitoring and biological-effect measures were collected in the same participants. • Duration of exposure was associated with DNA damage and protein oxidation; the observational design does not establish a causal dose–response relationship.
(34)	Lovreglio et al., 2014 — Italy	• 24 filling-station attendants. • 19 fuel-tanker drivers. • 31 controls; all male; Bari, Italy.	• Full ~8-h shift. • Passive personal sampler (Radiello®, active carbon cartridge).	• GC-FID (modified NIOSH method 1,501); LOD 3 μg/m^3^. • Urinary ttMA: HPLC-UV. • SPMA: HPLC-MS/MS. • Urinary benzene: headspace GC–MS.	• Airborne benzene (μg/m^3^): Filling-station attendants: mean 23.3 ± 17.0; median 19.9 (range 4.5–66.3). • Fuel-tanker drivers: mean 306.7 ± 266.7; median 246.6. • Controls: mean 4.6 ± 2.6; median 4.3. • Urinary biomarkers (attendants; median, μg/g Cr): SPMA: 0.56 (0.05–3.33) vs. controls 0.22 (*p* < 0.001). • ttMA: 87 (11–157). Urinary benzene: 0.24 μg/L (*p* < 0.001). • CA: 4.0 ± 1.9% vs. 3.7 ± 1.9% — NOT significant. • MN: 8.0 ± 3.0/1,000 BN — NOT significant. • MN significant in fuel-tanker DRIVERS only (*p* = 0.015).	• Air benzene in filling-station attendants was below occupational exposure limits but higher than in controls; SPMA and urinary benzene were also higher. • CA and MN frequencies were not significantly increased in filling-station attendants. • MN frequency was significantly increased in the more highly exposed fuel-tanker drivers; this group comparison does not establish a genotoxic threshold.
(31)	Neghab et al., 2015 — Iran	• 200 exposed PSA employees from all 20 private stations in Shiraz (194 M, 6F). • 200 unexposed controls. • Rotating 8-h shifts.	• 8-h personal breathing-zone samples. • Activated charcoal sorbent tubes (SKC No. 22601) + pump; 0.1–0.02 L/min. • 3 samples per station during different shifts (60 total).	• GC-FID (Varian CP-3800; 30 m × 0.25 mm capillary column). • LOD: 0.03 mg/m^3^ benzene.	• Personal BTX (mg/m^3^; geometric mean): Benzene: GM 0.8; mean 0.79; median 0.77; range 0.06–1.25 (TLV 1.6 mg/m^3^). • Toluene: GM 1.4; mean 1.47 (TLV 75 mg/m^3^). Xylene: GM 2.8; mean 3.0 (TLV 434 mg/m^3^). • ALT: 23.1 ± 13.9 vs. 19.4 ± 10.4 U/L (*p* = 0.018). • AST: 24.14 ± 9.2 vs. 22.3 ± 8.7 U/L (*p* = 0.04). • Serum albumin: 4.5 ± 0.5 vs. 4.8 ± 0.4 mg/dL (*p* < 0.001; lower in exposed). • All hepatorenal effects persisted after adjusting for age, smoking and BMI.	• None of the reported mean or maximum BTX concentrations exceeded the corresponding TLVs used in the study. • Differences in selected hepatic and renal indices were observed at sub-TLV concentrations; the cross-sectional findings do not establish causation. • This was the only study in the Table 2 selection to include female attendants (6/200; 3%).
(27)	Campo et al., 2016 — Italy	• 89 petrol station workers (all male; mixed: pump attendants, cashiers). • 90 controls. • Northwest Milan province; mean age 44 years.	• ~ 5-h mid-week shift. • Passive sampler (Radiello®, 35–50 mesh charcoal cartridge); LOQ 1.0 μg/m^3^. • Urine: Monday baseline, before sampling, end of sampling.	• GC–MS (Fustinoni method). • Urinary BTEX: HS-SPME-GC–MS; LOQ 15 ng/L. • Urinary SPMA and ttMA: LC–MS/MS. • Urinary cotinine: LC–MS/MS (LOQ 10 μg/L).	• Airborne BTEX in workers (μg/m^3^; median, 5th–95th percentile): Benzene: 59 (5–284); Toluene: 236 (24–1,763); Ethylbenzene: 23 (4–176); m + p-Xylene: 73 (11–608); MTBE: 408 (12–3,088). • Controls benzene: 4 (1–18) μg/m^3^. • Urinary benzene (BEN-U) at end of sampling: median 339 (90–2,749) ng/L vs. controls 157 (54–2,554) ng/L (*p* < 0.001). • SPMA: workers 0.19 μg/L vs. controls 0.10 μg/L. • ttMA: workers and controls comparable (NOT significantly different). • Biological exposure equivalents (non-smokers): BEN-U 1,457 ng/L ≡ EU OEL (1 ppm); 1,116 ng/L ≡ ACGIH TLV-TWA (0.5 ppm).	• Median personal benzene (59 μg/m^3^) was below the EU OEL (3,200 μg/m^3^) and ACGIH TLV-TWA (1,600 μg/m^3^). • Urinary benzene was more responsive than SPMA or ttMA at these low exposure levels. • ttMA was not significantly different between workers and controls. • The authors proposed urinary-benzene biological exposure equivalents for this occupational setting.
(55)	Moro et al., 2015 — Brazil	• 60 male GSAs (44 non-smokers, 16 smokers). • 28 non-smoking controls. • Rio Grande do Sul; mean exposure ~10 years.	Personal passive sampler (SKC 575–002®); ~8-h shift; CS₂ desorption.	• GC-FID (PerkinElmer; Innowax column). • Urinary ttMA: HPLC-UV (Ducos method). • HA and mHA: HPLC-UV; creatinine: spectrophotometry.	• Airborne benzene (μg/m^3^; median, IQR/range as reported for the cohort): 144.2 (58.1–2,207.5) in GSAs versus 42.0 (34.1–52.4) in controls (*p* < 0.001). • Median GSA benzene was below the ACGIH limit, but the upper reported range exceeded it. • Urinary ttMA (μg/g Cr; median): GSA nonsmokers 320.0; smokers 400.0; controls 70.0 (*p* < 0.001). • HA did not differ significantly; mHA was not detected. • CD80 and CD86 expression decreased; IL-8 increased; ALA-D activity decreased. • ttMA was associated with reduced CD80 (*β* = −0.278; *p* = 0.044) and CD86 (*β* = −0.379; *p* < 0.001).	• Higher ttMA and altered immune markers were observed in attendants. • ttMA was the exposure variable retained in models for reduced CD80 and CD86 expression. • These cross-sectional associations do not establish that ttMA itself caused the immune changes.
(56)	Moro et al., 2019 — Brazil	• 38 male GSAs. • 28 male controls. • Rio Grande do Sul.	Blood and urine sampling post-shift.	• Blood and urine BTX: GC–MS. • Oxidative stress markers. • Cytokines: ELISA.	• Blood benzene: significantly higher in GSAs vs. controls (*p* < 0.001). • Urinary ttMA significantly higher; urinary toluene 2 × higher. • Protein carbonyl significantly elevated; GST activity significantly reduced. • Pro-inflammatory cytokines (IL-8, TNF-α) significantly elevated. • Note: specific blood BTX concentrations shown in figures only — not reported as text values.	• Systemic BTX measurements, urinary biomarkers, oxidative-stress markers and inflammatory markers were assessed in the same participants. • Reduced GST activity was consistent with altered antioxidant defence in the exposed group. • Higher IL-8 and TNF-α were associated with occupational exposure; the cross-sectional study cannot establish a sustained inflammatory state caused specifically by benzene.
(26)	Scheepers et al., 2019 — Sri Lanka	• 29 male gas station attendants from 4 stations, Gampaha District. • 16 male office worker controls. • Measurements Jan–Apr 2018; long working hours (8–16 h/shift).	• Personal passive sampler (Radiello Orsa5 diffusive badge, 400 mg coconut shell coal) for full shift. • Active personal sampler (SKC Anasorb; 200 mL/min) in 25% of workers. • End-exhaled air: BioVOC sampler → ATD tube (pre- and post-shift, 3 days).	• GC-FID (Agilent 7890B; dual column; LOD 20 μg/m^3^ for 8-h sample). • End-exhaled air: TD-GC–MS. • Urinary SPMA: UPLC-MS/MS (Xevo TQ-XS; LOD 0.05 ng/mL).	• Breathing zone benzene (PPAS; median, range): 609 (65.1–1,960) μg/m^3^ in GSAs. • Toluene: 746 (<5.0–2,770) μg/m^3^; xylene isomers not detected by PPAS. • 28% of measurements exceeded ACGIH TLV-TWA (1,600 μg/m^3^) when adjusted for long working hours. • Highest task peaks (PID): refilling storage tank 239 ppm; measuring fuel level 96.4 ppm; routine refuelling 2.24 ppm. • End-exhaled air benzene: GSAs pre-shift 10.8 ng/L; post-shift 20.6 ng/L; 5–15-fold increase pre-to-post-shift (*p* < 0.005). • Urinary SPMA: GSAs pre-shift 2.40 μg/g Cr; post-shift 3.02 μg/g Cr; controls pre-shift 0.55 μg/g Cr. • Correlation personal benzene (PPAS) vs. exhaled benzene: *r* = 0.422 (*p* < 0.001).	• Personal air, end-exhaled air and urinary SPMA were measured concurrently. • Adjustment for long working hours resulted in 28% of measurements exceeding the ACGIH TLV-TWA. • Storage-tank refilling and fuel-level measurement produced the highest short-term peaks. • The moderate personal-air–exhaled-benzene correlation supports further evaluation of exhaled air as a non-invasive exposure indicator.

PSA, petrol station attendant; GSA, gasoline station attendant; M, male; F, female; GC-FID, gas chromatography–flame ionisation detection; GC–MS, gas chromatography–mass spectrometry; HS-SPME, headspace solid-phase microextraction; TD-GC–MS, thermal desorption GC–MS; HPLC-UV, high-performance liquid chromatography–UV detection; LC–MS/MS, liquid chromatography–tandem mass spectrometry; UPLC-MS/MS, ultra performance LC–tandem MS; OVM, organic vapour monitor badge; TWA, time-weighted average; TLV-TWA, ACGIH Threshold Limit Value–TWA; BEI, ACGIH Biological Exposure Index; OEL, occupational exposure limit; SPMA, S-phenylmercapturic acid; ttMA, trans,trans-muconic acid; BEN-U, urinary benzene; MHA, methylhippuric acid; HA, hippuric acid; MTBE, methyl tert-butyl ether; ALA-D, aminolevulinate dehydratase; IL-8, interleukin-8; TNF-α, tumour necrosis factor alpha; CA, chromosomal aberration; MN, micronucleus; BN, binucleated cells; SCE, sister chromatid exchange; HFC, high-frequency cells; OTM, Olive tail moment; LOD, limit of detection; LOQ, limit of quantification; PPAS, personal passive air sampler; PID, photoionisation detector; B/T, benzene-to-toluene ratio; GM, geometric mean; SD, standard deviation; IQR, interquartile range. Notes: Bahrami 2007 and 2008 — sampling duration was 2–4 h, recalculated to 8-h TWA. Moro 2019 — specific blood BTX concentrations shown in figures only. Periago and Prado 2005 — measures personal breathing-zone exposure and is placed in this table.

Scheepers et al. (26) implemented the most integrated design of the reviewed personal monitoring studies, simultaneously collecting personal air samples, end-exhaled air as a biological exposure proxy, and pre- and post-shift urinary SPMA. Post-shift exhaled BTX concentrations were 5–15-fold higher than pre-shift concentrations (*p* < 0.005); personal benzene correlated moderately with exhaled benzene (*r* = 0.422; *p* < 0.001); and pre-shift SPMA was higher in attendants than in controls. Fracasso et al. (28) combined personal monitoring, urinary biomarkers, and genotoxicity assessment in the same participants—one of the few studies spanning three domains—and found that comet assay tail moment correlated significantly with personal benzene exposure. Pandey et al. (29) measured air BTX by PID and blood BTX by GC–MS alongside comet assay and micronucleus testing, providing a rare three-domain data linkage.

Periago and Prado (30) demonstrated an important temporal finding: mean personal benzene exposure among Spanish service station attendants decreased from 736 μg/m^3^ in 1995 to 163 μg/m^3^ in 2003 following implementation of EU Directive 98/70/EC, which limited petrol benzene content to a maximum of 1% v/v from January 2000. This provides longitudinal evidence that regulatory control of fuel benzene content can reduce occupational exposure. Female PSA representation remains severely limited across the reviewed personal monitoring studies. Neghab et al. (31) was the only study in this selection to include female PSAs, with 6 of 200 exposed participants (3%) being female. Evidence from Santiago et al. (5) that female PSAs had higher chromosomal abnormality frequencies than males in the same cohort, and from Rekhadevi et al. (32) that 34 female PSAs had the highest SPMA concentrations among the reported subgroups, highlights an important evidence gap for sex-specific risk characterisation.

Smoking is an important potential confounder because tobacco smoke contributes to benzene exposure and can increase urinary benzene biomarkers, particularly at low occupational exposure levels (33). Approaches to smoking varied across the reviewed studies: some restricted participants to nonsmokers, some matched or statistically adjusted for smoking, and others measured cotinine, including Campo et al. (27) and Scheepers et al. (26). This heterogeneity limits cross-study comparisons at low benzene concentrations.

### 4.3 Biological monitoring of BTX exposure among PSAs

Table 3 presents a selection of biological monitoring studies reviewed for this synthesis. Urinary ttMA was the most frequently used biomarker, followed by urinary phenol, urinary benzene (U-BEN), SPMA, blood BTX, and urinary HA and MHA. As discussed in Section 3.5, ttMA has limited specificity at sub-ppm benzene concentrations because dietary sorbic acid can also increase urinary ttMA; SPMA and U-BEN are therefore preferred for low-level benzene exposure assessment. Among the reviewed studies, SPMA ranged from a median of 0.56 μg/g creatinine in Italian filling-station attendants (34) to a mean of 8.11 μg/L in Indian petrol station attendants (32). Because these values were expressed using different concentration bases, they should not be compared directly. Both were higher than their respective control values; only the creatinine-adjusted Lovreglio value can be compared directly with the ACGIH BEI of 25 μg/g creatinine, and it was below that value.

**Table 3:** Biological monitoring of BTX/BTEX exposure among petrol station attendants (PSAs) and comparable petroleum sector workers (*n* = 15 studies; 2001–2023).

Ref	Author, Year Country	Population (M/F)	Biological samples and analysis	Quantitative results	Interpretation
(57)	Wiwanitkit et al., 2001 — Thailand	• 30 male gas station attendants (non-smokers). • 49 male rural controls (non-smokers). • Ladprao district, Bangkok.	• Random urine collected 4–8 h after start of work shift. • Urinary ttMA by HPLC-UV (ion-exchange SPE; C-18 Lichrocart column; 1% acetic acid/methanol/sodium acetate mobile phase; UV detection; vanillic acid internal standard; Lee et al. modified method). • Creatinine by Jaffe reaction.	• Urinary ttMA (mean±SD; mg/g Cr): Gas station attendants: 4.00 ± 12.49 (range 0–64.2). • Controls: 0.116 ± 0.027 (range 0–0.40). • Significantly higher in attendants (*p* < 0.05). • One outlier with ttMA 64.2 mg/g Cr; excluding outlier, attendants mean 1.93 ± 5.66 mg/g Cr — still significantly elevated. • Atmospheric benzene at study sites: 0.76–4.14 ppm (contextual).	• Urinary ttMA significantly elevated in gas station attendants despite high variability. • The difference remained statistically significant after exclusion of the extreme value. • Urinary ttMA recommended as a practical biomarker for occupational benzene exposure in developing country settings where air monitoring is unavailable.
(35)	Fustinoni et al., 2005 — Italy	• Gasoline station attendants (*n* = 78). • Traffic policemen (*n* = 77). • Bus drivers (*n* = 152). • Milan referents/clerks (*n* = 58). • Genoa referents/clerks (*n* = 49). • Milan and Genoa, Italy; 35% overall smoking prevalence.	• Personal passive samplers worn during part of work shift. • End-of-shift urine spot samples. • Urinary ttMA by HPLC. • SPMA by HPLC-MS/MS. • U-benzene by GC/MS (Milan subjects only). • Urinary cotinine by immunoassay.	• Airborne benzene (μg/m^3^; median, min-max): Gasoline attendants: 61 (11–478); Traffic policemen: 22 (9–316); Bus drivers: 21 (<6–92); Milan referents: 6 (<6–115). • Urinary biomarkers — gasoline attendants (median, μg/L): ttMA non-smokers: 49 (<10–581); smokers: 144 (15–321). • SPMA non-smokers: 5.8 (0.2–10.9); smokers: 7.5 (0.2–24.8). • U-benzene non-smokers: 342 (42–2,836) ng/L; smokers: 1,168 (55–5,111) ng/L. • Smokers significantly higher than non-smokers for ALL biomarkers in all groups (*p* < 0.001). • U-benzene detectable at airborne benzene as low as 6 μg/m^3^ (0.002 ppm).	• Gasoline attendants had significantly higher airborne benzene than all other occupational groups (*p* < 0.01). • Smoking substantially confounds all urinary biomarkers — cotinine measurement essential in future studies. • U-benzene was detectable at airborne benzene concentrations as low as 6 μg/m^3^, supporting its usefulness for low-exposure monitoring. • ttMA and SPMA less sensitive at low exposures — SPMA more specific but smoking-affected.
(36)	• Fustinoni et al., 2010 — Italy • (J Chromatogr B 878:2534–2,540)	• 705 urine samples from 3 populations. • General urban population (*n* = 269). • Gasoline station attendants (*n* = 267). • Refinery petrochemical workers (*n* = 169). • Milan area, Italy; 2007–2008.	• U-benzene: headspace SPME-GC/MS (benzene-d6 internal standard; DB1 column 60 m × 0.25 mm × 1 μm; LOQ 15 ng/L). • SPMA: LC/MS/MS (SPMA-d2 internal standard; Betasil C18 column 150 mm × 2.1 mm; LOQ 0.1 μg/L). • Internal quality control: Shewhart charts, duplicate samples, two-laboratory SPMA comparison.	• Gasoline station attendants urinary biomarkers (median, 5th–95th percentile): • U-benzene non-smokers: 342 (42–2,836) ng/L; smokers: 1,168 (55–5,111) ng/L. • SPMA non-smokers: 5.8 (0.2–10.9) μg/L; smokers: 7.5 (0.2–24.8) μg/L. • ttMA non-smokers: 49 (<10–581) μg/L; smokers: 144 (15–321) μg/L. • U-benzene between-run precision: 10.3% at 120 ng/L; 4.8% at 1,000 ng/L. • SPMA between-run precision: 40.3% at 0.3 μg/L — less reliable near LOQ; acceptable at ≥1 μg/L. • Two-laboratory SPMA comparison: acceptable agreement (slope 0.863; Pearson *r* = 0.844).	• The U-benzene assay was reliable across the reported concentration range and suitable for low-exposure monitoring. • SPMA less reliable at concentrations below 1 μg/L — key methodological caveat for studies at low-level benzene exposures. • Studies reporting SPMA near the LOQ (0.1 μg/L) should state analytical performance explicitly. • Methodological paper that provides the quality control evidence base for U-benzene and SPMA as monitoring tools.
(32)	Rekhadevi et al., 2011 — India	• 200 fuel filling station attendants (166 M, 34F). • 200 matched controls. • Hyderabad; minimum 5 years employment; 8–10 h shifts. • Samples over 10 months.	• End-of-shift urine. • U-BZ by GC–MS (Agilent 6,890 + 5,973 MSD; 30 m × 250 μm × 0.25 μm; Waidyanatha method). • U-Ph by Khoschsorur and Peltek method. • ttMA by Ruppert et al. HPLC method. • SPMA by van Sittert et al. method.	• Overall mean urinary biomarker values (μg/L): U-BZ: FFSA 11.29 vs. controls 0.60 (*p* < 0.05). • U-Ph: FFSA 12.38 vs. controls 5.93 (*p* < 0.05). • ttMA: FFSA 145.06 vs. controls 40.90 (*p* < 0.05). • SPMA: FFSA 8.11 vs. controls 4.98 (*p* < 0.05). • By ambient air benzene exposure category (low/medium/high; μg/L, mean±SD): SPMA: 6.71 ± 0.74/8.32 ± 0.28/9.40 ± 0.41. • U-BZ: 8.89 ± 1.41/11.74 ± 0.54/13.44 ± 0.54 (significant graded increases for both). • SPMA correlated with CA (*r* = 0.30; *p* = 0.005) and MN (*r* = 0.23; *p* = 0.005). • ttMA correlated with CA (*r* = 0.18; *p* = 0.01) but NOT with MN (*p* = 0.37). • Blood benzene: FFSA 5.18 vs. controls 2.12 ppb (*p* < 0.05).	• Biomarker sensitivity rank at low benzene exposure: SPMA > U-BZ > U-Ph > ttMA. • SPMA and U-BZ increased across the ambient-air benzene exposure categories. • SPMA shows stronger correlation with genotoxicity endpoints (CA and MN) than ttMA — further supporting SPMA as the preferred biomarker. • ttMA was not correlated with MN frequency in this study and should not be treated as a genotoxicity surrogate on the basis of these data.
(58)	Tunsaringkarn et al., 2011 — Thailand	• 33 gasoline station workers (11 stations, Pathumwan district, Bangkok). • 30 office workers/students (controls). • April–June 2009; both sexes.	• Blood (2 mL) and urine (20 mL) collected 8 h after start of shift. • SCE by BrdU-Hoechst-Giemsa technique (15 metaphases per subject). • Urinary ttMA by modified HPLC-UV (Lee et al. method; Spherisorb ODS-2 column 150 mm × 4.6 mm; 259 nm; isocratic elution; vanillic acid internal standard). • Creatinine: LIASYS II analyser (Jaffe reaction).	• SCE frequency per cell (mean±SE): Gasoline workers: 13.47 ± 0.26 vs. controls 8.52 ± 0.40 (*p* < 0.01). • Chromosomal damage R*R* = 3.00 (95% CI 1.81–4.98; *p* < 0.001). • All 33 workers vs. 10/30 controls had SCE > 8.92/cell (control mean+SE cutoff). • Urinary ttMA (mean±SE): Workers: 1.74 ± 0.25 mg/L vs. controls 1.05 ± 0.29 mg/L (*p* < 0.05). • Workers ttMA/creatinine: 2.38 ± 0.63 vs. controls 1.03 ± 0.25 mg/g Cr (*p* < 0.05). • Sex difference: female workers ttMA significantly higher than male workers (2.39 ± 0.23 vs. 1.20 ± 0.37 mg/L; *p* < 0.05); female ttMA/creatinine 4.26 ± 1.23 vs. male 0.81 ± 0.15 mg/g Cr (*p* < 0.01). • Both control and worker ttMA/creatinine exceeded ACGIH BEI (0.5 mg/g Cr).	• SCE frequency significantly elevated — chromosomal damage risk 3 × higher in workers than controls. • Both groups exceeded the ACGIH BEI for ttMA; urban background exposure and other confounding sources should therefore be considered when interpreting the biomarker results. • Sex difference in ttMA warrants sex-stratified analysis in all PSA biomonitoring studies. • The findings are consistent with biological uptake of benzene in an urban high-background environment.
(54)	Moro et al., 2013 — Brazil	• 43 male GSAs (non-smokers). • 28 male controls. • Rio Grande do Sul, Brazil. • Mean exposure time 9.7 ± 1.1 years (range 0.5–32).	• Personal passive sampler (SKC 575–002®) for airborne BTX (~8 h). • End-of-shift urine and blood. • Urinary ttMA by HPLC-UV after SPE (Ducos method). • Comet assay (alkaline; silver nitrate staining; 100 cells; visual scoring 0–4). • Buccal cell MN assay (1,000 cells). • Oxidative stress: PCO, 3-NT, SOD, CAT, GST, GSH.	• Airborne BTEX (μg/m^3^; median, IQR): Benzene: 144.2 (58.1–2,207.5) GSA vs. 42.0 (34.1–52.4) controls (*p* < 0.001). • Toluene: 345.3 (213.0–3,945.1) vs. 107.2 (78.4–172.3). • o-Xylene: 182.0 (29.8–627.4) vs. 30.0 (21.6–65.3). Median concentrations were below the relevant ACGIH limits, although the upper benzene range exceeded the benzene limit. • Urinary ttMA: significantly higher in GSA vs. controls (*p* < 0.001); specific values shown in figures only. • DNA damage index (comet): significantly higher in GSA vs. controls. • Buccal cell MN frequency: significantly higher in GSA vs. controls. • Protein carbonyl and 3-nitrotyrosine: significantly higher in GSA. • Antioxidant enzymes (SOD, CAT, GST) and GSH: significantly lower in GSA. • Duration of benzene exposure correlated with DNA and protein damage.	• Multi-domain study: personal air monitoring, biological monitoring and biological-effect monitoring assessed in same subjects. • The lower central exposure estimates, together with some higher individual measurements, were associated with oxidative protein damage and genotoxicity. • Duration of exposure correlated with damage, supporting an exposure-related association; the cross-sectional design does not establish causality. • Reduced antioxidant enzyme capacity (SOD, CAT, GST) indicates oxidative stress mechanism.
(34)	Lovreglio et al., 2014 — Italy	• 24 filling-station attendants. • 19 fuel-tanker drivers. • 31 controls; all male; Bari, Italy.	• Full ~8-h shift passive sampler (Radiello®). • End-of-shift urine. • Urinary ttMA by HPLC-UV (264 nm; SAX SPE). • Urinary SPMA by HPLC-MS/MS. • Urinary benzene by headspace GC–MS. • Creatinine by DCA 2000 + colorimetric.	• Filling-station attendants — urinary biomarkers (median, range): • SPMA: 0.56 μg/g Cr (0.05–3.33); significantly higher than controls (*p* < 0.001). • ttMA: 87 μg/g Cr (11–157). • Urinary benzene: 0.24 μg/L (0.04–2.87); significantly higher than controls (*p* < 0.001). • Controls: SPMA 0.22 μg/g Cr (median); urinary benzene 0.12 μg/L. • Attendants airborne benzene: mean 23.3 ± 17.0 μg/m^3^; median 19.9 (range 4.5–66.3). • Fuel-tanker drivers significantly higher than attendants and controls for all biomarkers (*p* < 0.001). • CA: 4.0 ± 1.9% (attendants) vs. 3.7 ± 1.9% (controls) — NOT significant. • MN: 8.0 ± 3.0/1,000 BN vs. 7.3 ± 2.7 — NOT significant. • MN significantly dependent on airborne benzene in fuel-tanker drivers only (*p* = 0.015).	• SPMA and urinary benzene were elevated in attendants; the median airborne benzene concentration (19.9 μg/m^3^) was above the EU ambient-air limit of 5 μg/m^3^ but below occupational exposure limits. • CA and MN did not differ significantly between filling-station attendants and controls; these data do not establish a genotoxicity threshold. • The significant MN-airborne benzene association was observed in fuel-tanker drivers, the more highly exposed group. • Urinary benzene showed strong discrimination between exposed workers and controls at these exposure levels.
(59)	Goethel et al., 2014 — Brazil	• 43 male GSAs. • 34 male taxi drivers (TD). • 22 male non-exposed controls (NE). • Rio Grande do Sul; all non-smokers. • Mean exposure time GSA 11.03 ± 1.1 years.	• End-of-shift urine (after 3 consecutive days). • Urinary ttMA by HPLC-UV (Ducos method; expressed as μg/g Cr). • Urinary 8-OHdG by EIA kit (Cayman Chemicals; pg./g Cr). • Buccal cell MN (1,000 cells; Giemsa). • Comet assay (alkaline; silver nitrate; 100 cells; visual scoring 0–4). • COHb in whole blood by spectrophotometry (TD group only).	• Urinary ttMA: GSA significantly higher than NE (*p* < 0.001); positively correlated with time of exposure (*r* = 0.65; *p* < 0.001). • Specific ttMA mean values shown in boxplots — not stated as text values. • Urinary 8-OHdG: significantly higher in GSA than NE; correlated with comet TM (*r* = 0.439; *p* < 0.001), comet DI (*r* = 0.226; *p* < 0.05), and MN (*r* = 0.202; *p* < 0.05). • Specific 8-OHdG values shown in boxplots — not stated as text values. • COHb (taxi drivers vs. NE): NOT significantly different (*p* > 0.05). • ttMA not measured in taxi driver group.	• The ttMA-exposure duration correlation (*r* = 0.65) shows an association between biomarker level and years of work. • 8-OHdG correlated with comet and MN endpoints, supporting an association between oxidative DNA damage and genotoxicity measures. • COHb did not differ significantly between taxi drivers and controls in this sample.
(55)	Moro et al., 2015 — Brazil	• 60 male GSAs (44 non-smokers, 16 smokers). • 28 non-smoking controls. • Rio Grande do Sul, Brazil. • Mean exposure: 119.8 months (~10 years).	• Personal passive sampler (SKC 575–002®) for airborne BTX (~8 h). • End-of-shift urine. • Urinary ttMA by HPLC-UV (Ducos method). • Urinary HA and mHA by HPLC-UV simultaneously. • ALA-D activity. • CD80/CD86 expression by flow cytometry. • Serum IL-8 by ELISA. • Creatinine by spectrophotometry.	• Airborne BTEX (μg/m^3^; median, IQR): Benzene: 144.2 (58.1–2,207.5) GSA vs. 42.0 (34.1–52.4) controls (*p* < 0.001). The median benzene concentration was below the ACGIH limit, but the upper reported range exceeded it. • Urinary metabolites (μg/g Cr; median, IQR): ttMA — GSA non-smokers: 320.0 (190.0–450.0); smokers: 400.0 (300.0–600.0); controls: 70.0 (50.0–120.0; *p* < 0.001). • HA: no significant difference. mHA: not detectable in any group (below LOD 0.003 g/g Cr). • CD80: significantly decreased in GSA vs. controls (*p* < 0.05). • CD86: significantly decreased in GSA vs. controls (*p* < 0.05). • IL-8: significantly elevated in GSA vs. controls (*p* < 0.05). • ALA-D activity: significantly reduced in GSA vs. controls. • Multiple regression: ttMA only factor significantly contributing to CD80 reduction (*β* = −0.278; *p* = 0.044) and CD86 reduction (*β* = −0.379; *p* < 0.001). • Exposure time significantly contributed to ALA-D reduction (*β* = 0.752; *p* = 0.001).	• Elevated ttMA was consistent with benzene absorption; lower CD80/CD86 expression and higher IL-8 were observed in the exposed group. • In the regression analysis, ttMA significantly contributed to reduced CD80 and CD86 expression, supporting an association with the measured immune endpoints. • mHA was below the analytical detection limit in all groups. • Multi-domain study spanning personal monitoring, biological monitoring and immunological biological-effect assessment.
(60)	• Fenga et al., 2016 — Italy • [Same study population as Costa et al. 2016]	• 91 male gasoline station attendants (eastern Sicily). • 63 male office worker controls. • Mean lifetime benzene exposure 14.06 ± 6.23 years. • 9 smokers (9.9%) in exposed group.	• End-of-shift urine after 3 consecutive days of exposure. • Urinary ttMA by HPLC with diode array detection (Eureka Lab Division kit); μg/mL. • NF-κB p65, phospho-NF-κB p65, phospho-IκB-α, phospho-SAPK/JNK, phospho-p38 MAPK, phospho-STAT3 in PBMCs by PathScan® Inflammation ELISA (450 nm).	• Urinary ttMA (mean±SD): Gasoline attendants: 0.89 ± 0.57 μg/mL vs. controls 0.67 ± 0.45 μg/mL (*p* < 0.05). • NF-κB: 137.6% higher in exposed vs. controls (*p* < 0.001). • Phospho-IκB-α: 147.9% higher in exposed vs. controls (*p* < 0.001). • Phospho-STAT3: 32.4% DECREASE in exposed vs. controls (*p* < 0.001). • Phospho-p38 MAPK and SAPK/JNK: NOT statistically significant. • All protein concentrations except phospho-p38 positively and significantly correlated with ttMA levels. • Environmental benzene at pump sites ≤0.1 ppm (from station managers — not independently measured).	• NF-κB and IκB-α activation indicates pro-inflammatory signalling pathway upregulation at sub-OEL benzene exposures. • Lower phospho-STAT3 was observed in exposed workers; its clinical significance cannot be determined from this cross-sectional study. • Important limitation: environmental benzene derived from station manager reports, not independent measurement. • Signal-transduction measures correlated with ttMA, but the observational design and possible co-exposures do not establish a benzene-specific mechanism.
(61)	• Costa et al., 2016 — Italy • [Same study population as Fenga et al. 2016]	• 91 male gasoline station attendants (eastern Sicily). • 63 male office worker controls. • Mean lifetime benzene exposure 14.06 ± 6.23 years.	• End-of-shift urine after 3 consecutive days of exposure. • Urinary ttMA by HPLC with diode array detection (Eureka Lab Division kit); μg/mL. • ROMs by d-ROMS test (spectrophotometry 505 nm; Carr Units; Diacron International). • AOPP by spectrophotometry (340 nm; calibrated vs. chloramine-T; μmol/L). • AGE by fluorescence (350 nm/440 nm; AU/mL).	• Urinary ttMA (mean±SD): Gasoline attendants: 0.89 ± 0.57 μg/mL vs. controls 0.67 ± 0.45 μg/mL (*p* < 0.05). • ROMs: significantly higher in exposed vs. controls (*p* < 0.001); correlated with ttMA (*r* = 0.312; *p* = 0.0026). • AOPP: significantly higher in exposed vs. controls (*p* < 0.001). • AGE: NOT significantly different between groups. • Both ROMs and AOPP negatively correlated with fruit and vegetable consumption (*p* < 0.01). • Environmental benzene at pump sites ≤0.1 ppm (from station managers — not independently measured).	• AOPP was significantly higher in exposed workers, whereas AGE did not differ significantly between groups. • ROMs correlated with ttMA; this association does not by itself isolate benzene from other workplace exposures. • Diet (fruit and vegetable intake) is a significant modifier of oxidative stress markers — a confounder that should be measured in future studies. • Important limitation: environmental benzene not independently measured.
(37)	Geraldino et al., 2021 — Brazil	• 275 gasoline station workers: 94 convenience store workers (CSW; 70.2% female) + 181 filling station attendants (FSA; 89.0% male). • 119 office workers (OW; comparison). • 21 stations, Rio de Janeiro; 2015–2017.	• End-of-shift urine (3 work shifts, 8 h each). • Urinary HA (toluene metabolite) by HPLC-UV (Lichrosorb RP18 column 244 × 4 mm; 257 nm; methanol/acetic acid; LOD 0.0075 mg/L). • Urinary MHA (xylene metabolite) by HPLC-UV (238 nm; phosphate buffer/methanol 70:30; LOD 0.005 mg/L). • Creatinine by colorimetric endpoint (Bioclin®).	• Urinary biomarkers (mean±SD; g/g Cr): HA — CSW: 0.393 ± 0.308; FSA: 0.319 ± 0.271; OW: 0.083 ± 0.19 (*p* < 0.001). • MHA — CSW: 1.714 ± 1.333; FSA: 1.456 ± 1.266; OW: 0.016 ± 0.051 (*p* < 0.001). • CSW HA approximately 4.7 × higher than OW; FSA approximately 3.8 × higher. • MHA in GSWs approximately 100 × higher than OW. • 95th percentile MHA: CSW 4.977; FSA 3.996; OW 0.110 g/g Cr. • 92.6% CSW and 86.2% FSA had HA above OW mean cutoff. • 98.9% of both CSW and FSA had MHA above OW reference value. • Mean HA values were below the Brazilian BEI (2.5 g/g Cr). Mean MHA was above the 1.5 g/g Cr BEI in CSW (1.714) and slightly below it in FSA (1.456); the 95th percentiles exceeded the BEI in both groups. • CSW and FSA did NOT differ significantly from each other. • Significant symptoms in exposed vs. OW (*p* < 0.05): altered mood/depression, cramp, dizziness, drowsiness, weakness, weight loss.	• CSW and FSA had similar urinary HA and MHA despite different job tasks, suggesting shared environmental exposure and possible dermal exposure; the study does not quantify the dermal contribution. • The 95th-percentile MHA values exceeded the Brazilian BEI in both groups, indicating workers with comparatively high biomarker levels. • MHA approximately 100 × higher than OW provides strong evidence of occupational xylene absorption. • Several symptoms were more frequent in exposed workers, but the reported results do not establish that individual symptom levels correlated with the biomarkers.
(62)	da Poça et al., 2021 — Brazil	• 154 filling station attendants (FSA; 90.9% male). • 95 convenience store workers (CSW; 71.6% female). • 100 office workers (OW; comparison). • 21 stations, Rio de Janeiro; cross-sectional.	• End-of-shift urine. • Urinary ttMA by HPLC-UV (Shimadzu; 264 nm; Lichrosorb RP-18; Ducos method; mg/g Cr). • Comet assay (alkaline; 4 DNA damage classes). • CBMN assay (1,000 BN cells). • Immunophenotyping by flow cytometry (CD56+, CD3 + CD4+, CD3 + CD8+, CD45RA+, CD45RO+).	• Urinary ttMA (median, min-max; mg/g Cr): OW: 0.05 (<LOD–0.37); CSW: 0.10 (<LOD–6.20); FSA: 0.10 (<LOD–3.03). • Gas station workers approximately double OW (*p* = 0.012). • Comet assay: DNA damage Class 1 significantly higher in FSA and CSW vs. OW (*p* < 0.001). • CBMN assay (MN frequency; median, min-max): FSA: 7.00 (0–24.00) vs. CSW 5.00 (1.50–16.00) vs. OW 3.25 (0.50–13.50); FSA > OW and CSW (*p* < 0.001). • NBUD: significantly higher in both exposed groups vs. OW (*p* < 0.001). • CBPI: FSA higher than OW (*p* = 0.006). • Immunophenotyping: decreased CD3 + CD4 + T helper lymphocytes in FSA and CSW vs. OW. • Decreased naive T helper (CD45RA+) in FSA vs. OW. • Increased memory T helper (CD45RO+) in both exposed groups vs. OW. • Increased NK cells (CD56+) in FSA vs. OW and CSW. • Decreased CD4/CD8 ratio in FSA vs. OW (*p* < 0.05).	• Urinary ttMA was approximately twice as high in gas station workers as in office workers, consistent with greater benzene uptake. • MN frequency significantly elevated in FSA — genotoxic risk documented in same cohort with elevated biomarkers. • Immunophenotyping showed a lower CD4/CD8 ratio and higher NK-cell levels in FSA than in office workers; these findings were associated with occupational exposure but do not establish causation. • Multi-domain study: biological monitoring, genotoxicity and immunophenotyping in same subjects — one of the most comprehensive assessments reviewed.
(63)	Thetkathuek et al., 2022 — Thailand	• 100 fuel dispenser workers. • 100 workers away from dispensers (same stations). • Rayong Province; 8 stations; 200 total. • 68.5% female; mean age 30.25 ± 11.02 years; 66% smokers. • NOTE: No unexposed control group.	• End-of-shift urine (8-h shift). • HPLC-UV for all biomarkers: ttMA (benzene metabolite; μg/g Cr); Urinary toluene (TU; mg/L); Mandelic acid/MA (ethylbenzene metabolite; g/g Cr); Methylhippuric acid/MHA (xylene metabolite; g/g Cr).	• Fuel dispenser workers (mean±SD): ttMA: 449.28 ± 213.32 μg/g Cr; median 428.23 (95.58–1,202.56); GM 407.38. • TU: 0.0016 ± 0.002 mg/L; median 0.0010. • MA: 0.061 ± 0.012 g/g Cr; median 0.060 — below standard of 0.15 g/g Cr. • MHA: 0.43 ± 0.112 g/g Cr; median 0.41 — below BEI of 1.5 g/g Cr. • Workers away from fuel dispenser: ttMA: mean 413.17 ± 252.20 μg/g Cr; MHA: mean 0.40 ± 0.094 g/g Cr. • No significant difference between fuel dispenser workers and non-dispenser workers for any biomarker (*p* > 0.05). • Group mean and median biomarker values were below the cited BEIs; however, the individual ttMA range extended to 1,202.56 μg/g Cr, above the 500 μg/g Cr BEI. • Significant predictors of ttMA (*p* < 0.05): male sex; low BMI; work >8 h/day; overtime ≥6 h/week. • Significant predictor of TU: ineffective personal hygiene. • Significant predictor of MA: absence of PPE.	• No significant biomarker differences were found between dispenser and non-dispenser workers; similar within-station exposure is possible, but the absence of an unexposed control prevents confirmation. • Group means and medians were below the cited BEIs, although some individual ttMA results exceeded the BEI. • Longer work hours predicted ttMA, ineffective hygiene predicted urinary toluene, and absence of PPE predicted mandelic acid; these are potentially modifiable workplace factors. • Absence of unexposed control group is a methodological limitation — comparisons are within-station only.
(64)	Giardini et al., 2023 — Brazil	• 324 gas station workers (GSW; all roles: attendants, cashiers, managers, convenience store). • 218 office workers (OW; not occupationally exposed). • 21 stations, Rio de Janeiro; 2014–2016. • Both sexes; 69.4% male among GSW.	• End-of-shift urine (3 work shifts, 8 h each). • Urinary ttMA by HPLC-UV (Ducos method modified; Luna Phenomenex® column 250 × 4.6 mm; LOD 0.00075 mg/L; LOQ 0.0013 mg/L). • Urinary HA by HPLC-UV (Lichrosorb RP18). • Urinary MHA by HPLC-UV (Lichrosorb RP18). • Full blood count by automated analyser (Sysmex XE-2100).	• Urinary biomarkers (mean±SD): ttMA — GSW: 0.28 ± 0.62 mg/g Cr vs. OW 0.13 ± 0.22 mg/g Cr (*p* = 0.001). • HA — GSW: 0.50 ± 0.29 g/g Cr vs. OW 0.08 ± 0.19 g/g Cr (*p* < 0.001). • MHA — GSW: 1.57 ± 1.44 g/g Cr vs. OW 0.02 ± 0.05 g/g Cr (*p* < 0.001). • Serial collection workers (SCW; *n* = 17): ttMA 0.23 ± 0.23; HA 0.40 ± 0.31; MHA 1.73 ± 1.42 g/g Cr. • Confounding variables (smoking, alcohol, processed food): no significant influence on biomarker levels. • Haematological findings: GSW differed significantly from OW for erythrocytes, haemoglobin, haematocrit, neutrophils, and lymphocytes. • 5 workers with persistent leukocytosis across 3 serial collections (15-day intervals): total leukocytes 10,300–12,160/μL; lymphocyte percentages consistently low (21.8–32.3%). • Most reported symptoms in GSWs: somnolence (45.1%); headache (38.3%); dizziness (27.5%); tingling (25.4%); involuntary movement (25.0%).	• MHA approximately 78 × higher in GSW than OW — striking evidence of occupational xylene absorption across all gas station worker roles. • Persistent leukocytosis in five workers across serial collections warrants clinical follow-up, but its cause cannot be determined from this study. • Haematological differences were observed between GSW and OW despite most values remaining within reference ranges; their clinical significance requires further study. • Smoking, alcohol intake and processed-food consumption were not significantly associated with biomarker levels in the reported analysis; residual confounding remains possible. • Largest Brazilian biomonitoring cohort in this review (*n* = 324 workers).

Studies ordered chronologically. Fustinoni 2005 and Fustinoni 2010 are separate publications: the 2005 study (Cancer Epidemiol Biomarkers Prev 14:2237–2,244) reports comparative biomarker data across occupational groups, while the 2010 study (J Chromatogr B 878:2534–2,540) evaluates analytical method quality. Costa et al. (61) and Fenga et al. (60) report data from the same 91 exposed + 63 control subjects in eastern Sicily. U-BZ/U-BEN, urinary benzene; ttMA, trans,trans-muconic acid; SPMA, S-phenylmercapturic acid; U-Ph, urinary phenol; HA, hippuric acid; MHA, methylhippuric acid; MA, mandelic acid; TU, urinary toluene; ROMs, serum reactive oxygen metabolites; AOPP, advanced oxidation protein products; AGE, advanced glycation end-products; ALA-D, aminolevulinate dehydratase; IL-8, interleukin-8; 8-OHdG, 8-hydroxydeoxyguanosine; PCO, protein carbonyl; 3-NT, 3-nitrotyrosine; SOD, superoxide dismutase; CAT, catalase; GST, glutathione S-transferase; GSH, glutathione; NF-κB, nuclear factor kappa B; STAT3, signal transducer and activator of transcription 3; p38-MAPK, p38 mitogen-activated protein kinase; PBMCs, peripheral blood mononuclear cells; MN, micronucleus; CBMN, cytokinesis-block micronucleus; NBUD, nuclear bud; CBPI, cytokinesis-block proliferation index; BN, binucleated cells; BEI, ACGIH Biological Exposure Index; M, male; F, female; GM, geometric mean; SD, standard deviation; IQR, interquartile range; HPLC-UV, high-performance liquid chromatography–UV detection; GC–MS, gas chromatography–mass spectrometry; SPE, solid-phase extraction; LOD, limit of detection; LOQ, limit of quantification.

Among 200 Indian petrol station attendants, Rekhadevi et al. (32) reported the following sensitivity ranking for the assessed benzene biomarkers: SPMA > U-BE*N* > urinary phenol > ttMA. Fustinoni et al. (35) demonstrated that U-BEN could detect exposure at airborne benzene concentrations as low as approximately 6 μg/m^3^, a concentration at which ttMA has limited reliability. In a separate analytical-method study, Fustinoni et al. (36) reported between-run imprecision of 40.3% for SPMA at 0.3 μg/L, close to the assay’s LOQ of 0.1 μg/L, whereas performance was acceptable at concentrations of at least 1 μg/L. This is an important limitation when interpreting SPMA results close to the detection limit.

Potential confounders and alternative exposure routes were handled inconsistently across the reviewed studies. Smoking is particularly important for U-BEN and SPMA, while dietary sorbic acid can confound ttMA; however, these factors were not consistently assessed or controlled. Dermal exposure was also rarely examined. Geraldino et al. (37) found similarly elevated urinary HA and MHA in convenience-store workers and filling-station attendants compared with office workers. Because convenience-store workers may still share the station environment, these findings suggest possible dermal and/or shared environmental exposure rather than proving dermal absorption alone. Ghahri et al. (38) used benchmark-dose modelling and reported a lower confidence limit (BMDL) of 0.12 ppm personal benzene for an oxidative DNA-damage endpoint. This finding supports further evaluation of health effects at concentrations below the current ACGIH TLV-TWA, but a single cross-sectional benchmark estimate should not be used alone to define an occupational exposure limit.

### 4.4 Biological-effect monitoring among PSAs

Supplementary Table 1 presents a selection of biological monitoring studies reviewed for this synthesis, grouped by category. The predominant limitation across all sub-domains is the absence of concurrent BTX exposure measurement: fewer than 30% of biological-effect monitoring studies included quantitative BTX exposure data, making it impossible to establish dose–response relationships from these studies alone.

#### 4.4.1 Genotoxicity

Genotoxicity is the most extensively studied biological effect of BTX exposure among PSAs, reflecting the Group 1 carcinogenic classification of benzene (3). Santos-Mello and Cavalcante (16) documented the first cytogenetic evidence in Brazilian gas station attendants, demonstrating significantly elevated chromosome deletions (0.829% vs. 0.126% in controls; *p* < 0.05) with a sampling context of particular concern — attendants had to place their ear near the tank opening to detect fullness during fuelling in 1984. Pandey et al. provided a multi-domain dataset documenting air BTX (100–250 ppb at pump stations), blood benzene (mean 7.94 ppb in workers vs. 2.82 ppb in controls; *p* < 0.05), comet assay (OTM 4.14 ± 1.25 vs. 3.29 ± 0.92; *p* < 0.001), and micronucleus frequency (14.79 ± 3.92% vs. 7.54 ± 3.00%; *p* < 0.001) in the same individuals — rare three-domain linkage (29). Martinez-Valenzuela et al. (39) documented elevated micronuclei (6.6 ± 0.5 vs. 1.8 ± 0.2 per 3,000 cells; FI = 3.66; *p* = 0.001) and chromatin condensation (164.1 ± 16.8 vs. 11.3 ± 1.5; FI = 14.52; *p* = 0.001) in Mexican PSAs, with no additional significant effect of smoking, alcohol or years worked — identifying gasoline vapour exposure as the primary genotoxic driver. Fracasso et al. (28) demonstrated a direct correlation between personal benzene, urinary SPMA, and comet assay tail moment in the same individuals (*r* = 0.509; *p* = 0.007), providing strong three-domain mechanistic linkage at sub-OEL concentrations.

Shaikh et al. (40) used the cytokinesis-block MN assay to document MN frequency of 17.39 ± 0.58 vs. 6.09 ± 0.40 per 1,000 binucleate cells (*p* < 0.001) in Indian PSAs with 15–20 years of exposure, alongside significantly elevated SCE (85.63 ± 2.15 vs. 75.80 ± 1.85; *p* < 0.001) and reduced CCPI (1.93 ± 0.03 vs. 2.33 ± 0.01; *p* < 0.001), indicating reduced proliferative capacity. *In vitro* supplementation with vitamin C (20 mM) and vitamin E (10 mM) significantly ameliorated all endpoints but did not restore them to control levels, suggesting that antioxidant supplementation may partially protect against petrol-induced genotoxicity (40). The epidemiological significance is underscored by Schnatter et al. (41) international pooled analysis, which found myelodysplastic syndrome significantly associated with benzene exposure in petroleum workers (highest tertile: O*R* = 4.33; 95% CI 1.31–14.3; *p* = 0.03), with a monotonic dose–response for cumulative benzene exposure (*p* trend = 0.01).

#### 4.4.2 Haematological and biochemical effects

Benzene is a well-established haematotoxin via its metabolites’ selective toxicity to bone marrow progenitor cells. Udonwa et al. (42) in Nigeria demonstrated that mean MetHb was 5.8% in PSAs vs. 2.7% in controls, and PCV was 30.8% vs. 40.8%, with a clear duration-response: MetHb rose from 1.8% at <1 year exposure to 13.6% at >10 years. Nwanjo and Ojiako (43) established a duration-response threshold for hepatorenal toxicity in Nigerian PSAs: no significant differences from controls at 1–5 years, but significantly elevated AST (56.82 ± 3.64 vs. 32.26 ± 2.68 IU/L), ALT (48.2 ± 3.35 vs. 30.8 ± 2.9 IU/L), creatinine (1.6 ± 0.36 vs. 0.98 ± 0.21 mg/dL), and proteinuria at ≥6 years exposure. Neghab et al. (31) documented the same hepatorenal effects at personal benzene concentrations below the ACGIH TLV-TWA, providing rare concurrent exposure-effect data: all BTX values were sub-TLV yet ALT, AST, serum albumin, total protein, urea, creatinine, and serum calcium were all significantly affected. Abou-ElWafa et al. (44) in Egypt found that RBC (4.8 vs. 5.3 million/mcL), Hb (13.9 vs. 15.2 g/dL), and HCT (39.7% vs. 43.7%) were all significantly reduced in 102 Egyptian PSAs vs. controls, with ALT being the only significantly elevated biochemical parameter.

#### 4.4.3 Oxidative stress and immunological effects

Toson et al. (45) established that prolonged gasoline exposure promotes M1 macrophage polarisation via oxidative stress, documenting significantly elevated TNF-α (186.7 ± 20 vs. 134 ± 19 ng/100 mL; *p* < 0.05), TNF-α/AMCase ratio (9.5 ± 0.6 vs. 5.44 ± 1.14; *p* < 0.05), reduced catalase (372.5 ± 30.9 vs. 481.1 ± 43.5 U/L; *p* < 0.01), reduced GSH (22.76 ± 1.6 vs. 30.76 ± 8.6 mg/dL; *p* < 0.001), and elevated MDA (7.794 ± 0.613 vs. 6.453 ± 0.067 μmol/mL packed cells; *p* < 0.05) in Egyptian GSAs vs. controls. Xia et al. (23) provided the strongest integrated evidence in this domain, combining environmental BTEX measurement with a comprehensive oxidative stress panel in the same workers: SOD was significantly lower (39.18 ± 6.05 vs. 52.84 ± 3.87 U/mL; *p* < 0.001) and MDA significantly higher (1.68 ± 0.52 vs. 1.43 ± 0.64 nmol/L; *p* = 0.021) in refuelling workers vs. office controls, despite all BTEX values remaining below Chinese national occupational standards. Ahmadi et al. (46) documented paradoxically elevated Hb (16.76 ± 0.14 vs. 15.25 ± 0.14 g/dL; *p* < 0.001), HCT, and RBC in Iranian GSAs — attributed to compensatory secondary polycythaemia in response to chronic MetHb-induced functional anaemia — alongside significantly elevated MetHb (1.07 ± 0.07 vs. 0.39 ± 0.04 g/dL), haemichrome (0.80 ± 0.07 vs. 0.37 ± 0.02 g/dL), and protein carbonyl (7.47 ± 0.33 vs. 5.81 ± 0.19 mmol/mg protein; all *p* < 0.001).

#### 4.4.4 Pulmonary function

Three Indian studies consistently documented reduced spirometric indices in petrol pump workers. Singhal et al. (9) found FVC (3.00 ± 0.75 vs. 3.96 ± 0.34 L; *p* < 0.05) and FEV₁ (2.65 ± 0.67 vs. 3.57 ± 0.41 L; *p* < 0.05) both significantly reduced, with FEV₁/FVC ratio NOT significantly different — a pattern of restrictive impairment — alongside reduced flow rates (PEFR, FEF₂₅₋₇₅, PIFR, FIF₅₀ all *p* < 0.05), suggesting small airway involvement. Solanki et al. (6) confirmed these findings in a larger cohort of 227 non-smoking petrol pump workers vs. 227 matched controls, with all spirometric parameters significantly reduced and a progressive worsening pattern in workers with >10 years exposure, progressing from restrictive to mixed (restrictive + obstructive) impairment. None of these studies included concurrent BTX exposure measurements, precluding quantitative dose–response characterisation.

### 4.5 Cross-domain mapping

Table 4 provides a comprehensive mapping of all included studies across the four EPHP monitoring domains. Studies with a tick (√ green shading) in a given column contribute quantitative data to that monitoring domain; a dash (—) indicates the domain was not assessed. No single study addressed all four domains simultaneously.

**Table 4:** Cross-domain mapping of all included studies across the four environmental public health paradigm (EPHP) monitoring domains.

Ref	Author, Year, Country	Environmental monitoring	Personal monitoring	Biological monitoring	Biological-effect monitoring
Studies contributing primarily to environmental monitoring (Table 1)					
(20)	Gonzalez-Flesca et al., 2002 — France	✓	—	—	—
(21)	Tunsaringkarn et al., 2012 — Thailand	✓	—	—	—
(17)	Zoleikha et al., 2017 — Iran	✓	✓	—	—
(23)	Xia et al., 2017 — China	✓	—	—	✓
(19)	Chaiklieng, 2021 — Thailand (PLoS ONE)	✓	—	—	—
(22)	Salama et al., 2021 — Saudi Arabia	✓	—	—	—
(18)	Allahabady et al., 2022 — Iran	✓	—	—	—
(47)	Costa-Amaral et al., 2019 — Brazil	✓	—	✓	✓
Studies contributing primarily to personal monitoring (Table 2)					
(24)	Lagorio et al., 1993 — Italy	—	✓	—	—
(48)	Carere et al., 1995 — Italy	—	✓	—	✓
(49)	Pitarque et al., 1997 — Spain	—	✓	—	✓
(50)	Romieu et al., 1999 — Mexico	—	✓	✓	—
(30)	Periago and Prado, 2005 — Spain	—	✓	—	—
(51)	Raghavan and Basavaiah, 2005 — India	—	✓	✓	—
(25)	Bahrami et al., 2007 — Iran (benzene/ttMA)	—	✓	✓	—
(52)	Bahrami et al., 2008 — Iran (xylene/MHA)	—	✓	✓	—
(53)	Keretetse et al., 2008 — South Africa	—	✓	—	✓
(29)	Pandey et al., 2008 — India	—	✓	✓	✓
(28)	Fracasso et al., 2010 — Italy	—	✓	✓	✓
(54)	Moro et al., 2013 — Brazil	—	✓	✓	✓
(34)	Lovreglio et al., 2014 — Italy	—	✓	✓	✓
(31)	Neghab et al., 2015 — Iran	—	✓	—	✓
(27)	Campo et al., 2016 — Italy	—	✓	✓	—
(55)	Moro et al., 2015 — Brazil	—	✓	✓	✓
(56)	Moro et al., 2019 — Brazil	—	✓	✓	✓
(26)	Scheepers et al., 2019 — Sri Lanka	—	✓	✓	—
Studies contributing primarily to biological monitoring (Table 3)					
(57)	Wiwanitkit et al., 2001 — Thailand	—	—	✓	—
(35)	Fustinoni et al., 2005 — Italy	—	✓	✓	—
(36)	Fustinoni et al., 2010 — Italy	—	—	✓	—
(32)	Rekhadevi et al., 2011 — India	—	—	✓	✓
(58)	Tunsaringkarn et al., 2011 — Thailand	—	—	✓	✓
(59)	Goethel et al., 2014 — Brazil	—	—	✓	✓
(60)	Fenga et al., 2016 — Italy	—	—	✓	✓
(61)	Costa et al., 2016 — Italy	—	—	✓	✓
(37)	Geraldino et al., 2021 — Brazil	—	—	✓	—
(62)	da Poça et al., 2021 — Brazil	—	—	✓	✓
(63)	Thetkathuek et al., 2022 — Thailand	—	—	✓	—
(64)	Giardini et al., 2023 — Brazil	—	—	✓	✓
(38)	Ghahri et al., 2024 — Iran	—	✓	—	✓
Studies contributing primarily to biological-effect monitoring (Table 4)					
(16)	Santos-Mello and Cavalcante, 1992 — Brazil	—	—	—	✓
(65)	Jakobsson et al., 1993 — Sweden	—	—	—	✓
(66)	Rushton and Romaniuk, 1997 — UK	—	—	—	✓
(41)	Schnatter et al., 2012 — International	—	—	—	✓
(42)	Udonwa et al., 2009 — Nigeria	—	—	—	✓
(67)	Bindhya et al., 2010 — India	—	—	—	✓
(68)	Sellappa et al., 2010 — India	—	—	—	✓
(69)	Mahmood, 2012 — Iraq	—	—	—	✓
(70)	Beerappa et al., 2013 — India	—	—	—	✓
(5)	Santiago et al., 2014 — Brazil	—	—	—	✓
(44)	Abou-ElWafa et al., 2015 — Egypt	—	—	—	✓
(71)	Metgud et al., 2015 — India	—	—	—	✓
(72)	Naidoo et al., 2016 — South Africa	—	—	—	✓
(39)	Martinez-Valenzuela et al., 2017 — Mexico	—	—	—	✓
(73)	Salih et al., 2019 — Iraq	—	—	—	✓
(46)	Ahmadi et al., 2019 — Iran	—	—	—	✓
(45)	Toson et al., 2022 — Egypt	—	—	—	✓
(40)	Shaikh et al., 2021 — India	—	—	—	✓
(43)	Nwanjo and Ojiako, 2007 — Nigeria	—	—	—	✓
(74)	Ajugwo et al., 2014 — Nigeria	—	—	—	✓
(9)	Singhal et al., 2007 — India	—	—	—	✓
(7)	Madhuri and Chandrashekar, 2012 — India	—	—	—	✓
(6)	Solanki et al., 2015 — India	—	—	—	✓
(75)	Filho et al., 2018 — Brazil	—	—	—	✓

✓ (green shading), study contributes quantitative data to this domain. —, domain not assessed in this study. Studies are grouped by their primary contribution; studies contributing to multiple domains are identified by ticks across the relevant columns. This table illustrates that no single published study has simultaneously implemented all four monitoring domains in a single petrol station attendant (PSA) cohort — a notable evidence gap identified in this review. EPHP, Environmental Public Health Paradigm; PSA, petrol station attendant; BTX, benzene, toluene, xylene; BTEX, benzene, toluene, ethylbenzene, xylene; Environmental monitoring, measurement of BTX/BTEX concentrations in ambient air at or near the worksite; Personal monitoring, measurement of BTX/BTEX in the individual worker’s breathing zone using personal samplers; Biological monitoring, measurement of BTX/BTEX compounds or metabolites (urinary ttMA, SPMA, U-benzene, HA, MHA, blood benzene) reflecting internal absorbed dose; Biological-effect monitoring, measurement of early adverse biological responses including genotoxicity, haematotoxicity, oxidative stress, immunological changes, and pulmonary function impairment.

## 5 Discussion

Before presenting the synthesised findings, one conceptual clarification is warranted. Biological-effect monitoring is not universally regarded as part of exposure assessment in the classical occupational hygiene sense. Personal monitoring and biological monitoring are established approaches to exposure assessment and do not require simultaneous biological-effect monitoring. In this review, biological-effect monitoring is included as a downstream component of the exposure–dose–effect continuum to facilitate discussion of early biological responses associated with BTX exposure and absorption; it is not intended to redefine exposure assessment more broadly than it is conventionally understood.

This narrative review synthesises three decades of BTX exposure assessment evidence among PSAs across four monitoring domains. Three central conclusions emerge. First, BTX concentrations were generally higher at refuelling worksites than at comparison locations, and some studies in LMIC settings reported benzene concentrations above the cited OELs. Reported concentrations varied widely across sampling designs, from low microgram-per-cubic-metre levels to more than 5 ppm at some ambient sampling points. Second, biological monitoring confirms systemic benzene absorption, with SPMA and U-BEN among the most sensitive and specific biomarkers; urinary exposure biomarkers were generally higher in PSAs than in comparison groups. Third, biological-effect monitoring studies report genotoxic, haematological, oxidative-stress and immunological alterations in PSA populations across several countries, although findings and methods were not uniform across all studies.

The reviewed studies were distributed across Europe, Asia, Latin America and Africa, with many conducted in LMIC settings. Sub-Saharan Africa contributed five reviewed studies—three from Nigeria and two from South Africa. Of these, Keretetse et al. (53) quantified personal BTX exposure in South African workers, whereas the three Nigerian studies assessed biological effects without directly measuring BTX in ambient air, personal breathing zones or biological specimens. This remains an important evidence gap for Nigeria, where the size of the petrol-retailing sector implies a substantial workforce with incompletely characterised exposure. Several personal-monitoring studies from LMIC settings—notably India, Sri Lanka and Iran—reported higher personal benzene concentrations than studies from Italy and Spain; however, differences in sampling methods, periods and workplace conditions limit direct regional comparisons. The observed variation may reflect differences in fuel composition, engineering controls, work practices and regulatory enforcement.

The preponderance of studies using ttMA as the primary biological monitoring endpoint—rather than the more specific SPMA and U-BEN—reflects a historical preference for analytically simpler methods. Fustinoni et al. (36) demonstrated that SPMA and U-BEN provide greater specificity and sensitivity than ttMA at low-level benzene exposure, and the other reviewed biomonitoring studies support their usefulness. Future integrated PSA monitoring studies should prioritise SPMA and U-BEN as primary benzene biomarkers and measure urinary cotinine to assess and control confounding from tobacco smoke. The analytical quality-control data of Fustinoni et al. (36) should also be noted: SPMA assay precision is reduced near the LOQ (0.1 μg/L), and studies reporting SPMA at very low levels should state their analytical performance explicitly.

A notable finding of this review is that, to our knowledge, no published study identified in this review has simultaneously implemented all four monitoring domains in a single PSA cohort. The closest approximations are Scheepers et al. (26), who combined personal monitoring, exhaled air, and urinary SPMA but omitted biological-effect monitoring; Fracasso et al. (28), who combined personal monitoring, urinary biomarkers, and CA assessment but omitted dedicated environmental monitoring; and Pandey et al. (29), who combined personal and blood BTX with comet assay and MN — spanning three domains but lacking environmental monitoring. Costa-Amaral et al. (47) came closest to four-domain integration (ambient air monitoring + urinary biomarkers + multiple genotoxicity and oxidative stress endpoints) but lacked personal monitoring data. This gap limits dose–response inference and the development of evidence-based occupational health policy.

It is important to emphasise that the absence of integrated four-domain studies does not negate the existing evidence supporting strengthened preventive action. Reports of benzene concentrations above cited OELs in some LMIC settings, together with multiple studies reporting genotoxic, haematological and oxidative-stress alterations, support measures such as vapour-recovery systems, exposure monitoring, appropriate gloves and work practices, worker training, and risk-based occupational health surveillance. Regulatory bodies should not interpret the absence of integrated four-domain studies as a reason to defer proportionate preventive measures. Integrated studies would additionally provide empirical cross-domain correlation data—for example, the relationship between personal air concentrations, internal-dose biomarkers and biological-effect endpoints within the same workers. Such evidence could help determine how well a single-domain approach reflects other points on the exposure–dose–effect continuum and guide the selection of feasible monitoring strategies in resource-constrained settings.

Methodological limitations that recur across the reviewed literature include predominantly cross-sectional designs; single-day sampling in several studies; inconsistent assessment and control of smoking; limited assessment of dermal exposure, although skin contact may contribute to total exposure among workers who handle fuel without suitable gloves; predominance of male study populations; and absent or retrospective exposure assessment in many biological-effect studies, limiting dose–response characterisation. Future studies should consider longitudinal or repeated-measures designs, include both sexes with sex-stratified analyses where adequately powered, measure urinary cotinine and account for dietary sorbic acid when ttMA is used, and apply validated, fit-for-purpose analytical methods with clearly reported quality-control performance.

## 6 Conclusion

This narrative review indicates that petrol station attendants are an occupational group with potential for chronic BTX exposure. Concentrations were generally higher at refuelling worksites and among attendants than at comparison locations or in comparison groups, and some studies in LMIC settings reported benzene concentrations above the cited OELs. Personal benzene concentrations ranged from below applicable limits in regulated European settings to above cited limits in some LMIC studies; ambient monitoring also identified high concentrations at some sampling points. Because sampling designs and workplace conditions differed substantially, the variation between studies cannot be attributed solely to differences in engineering controls or regulatory enforcement. Biological-effect studies reported genotoxic, haematological, hepatorenal, oxidative-stress, immunological and pulmonary-function alterations, although the predominantly cross-sectional designs and frequent absence of concurrent BTX measurements limit causal attribution.

To our knowledge, no published study identified in this review simultaneously applied all four EPHP monitoring domains in a single PSA cohort using standardised protocols and systematic confounder control. SPMA and U-BEN are among the preferred sensitive and specific biomarkers for biological monitoring of low-level benzene exposure. The comet assay, micronucleus frequency and 8-OHdG may provide complementary biological-effect information, while urinary cotinine is recommended to assess and control confounding from tobacco smoke.

The limited integrated BTX monitoring evidence from sub-Saharan Africa—and Nigeria specifically—constitutes an important evidence gap with policy implications. Further well-designed studies in Nigerian and broader African PSA cohorts are recommended. In resource-constrained LMIC settings, studies using validated personal-air or biological-monitoring methods with clearly reported analytical quality control would be informative. Where resources permit, integrated multi-domain designs could quantify relationships across the exposure–dose–effect continuum and help determine the most informative feasible monitoring strategies for subsequent studies. Such evidence could support proportionate policies on vapour-recovery systems, exposure and health surveillance, worker training, appropriate protective equipment and safer fuel-handling practices at petrol stations.
