# Supplementary material for: Exposure assessment of benzene, toluene, and xylene in petrol station attendants: methods, biomarkers, and the case for integrated monitoring: a narrative review
Source: Front Public Health. 2026 Oct 14;14:1920988. doi: 10.3389/fpubh.2026.1920988 (PMC13714498; doi:10.3389/fpubh.2026.1920988)
Supplement: Supplementary file 1 [file Table_1.docx]

**Supplementary Table 1 | Biological-effect monitoring among petrol station attendants (PSAs) and comparable petroleum sector workers, grouped by endpoint category (n=32 studies; 1992–2021)**

| **Ref** | **Author, Year Country** | **Population (M/F)** | **Biological Effect Assessed** | **Concurrent Exposure**  **Measured?** | **Smoking**  **Addressed?** | **Quantitative Results** | **Interpretation** |
| --- | --- | --- | --- | --- | --- | --- | --- |
| **GROUP A — GENOTOXICITY** | | | | | | | |
| [16] | - Santos-Mello & - Cavalcante,   1992   - Brazil | • 49 male GSAs from 9 stations in Rio de Janeiro and São Paulo. • 24 male controls. • Sampled July– Nov 1984. | - CA in PBL: chromosome   deletions, chromatid aberrations, chromatid gaps, aneuploid cells, dicentrics.   - 150 metaphases per subject; Giemsa staining; coded slides. | No | Analysed by smoking status within workers | - Chromosome deletions — workers:   0.829±0.112% vs controls  0.126±0.056%  (Z=2.266; p<0.05)  — ONLY  SIGNIFICANT endpoint.   - 46.9% of workers vs 16.7% of controls had ≥1   chromosome aberration (χ²=5.162; p<0.05).   - Chromatid gaps, chromosome gaps, aneuploid cells, dicentrics: NOT significantly different. - Smoking had NO   significant effect on any CA endpoint within the exposed group.   - No significant correlation between   chromosome  deletions and age or duration of employment. | - First study to document cytogenetic damage specifically in gas station attendants. - Context: in 1984, attendants placed ear near tank opening to detect fullness during fuelling — direct fume inhalation. - Chromosome deletions specifically elevated — a recombinogenic signature consistent with benzene metabolite activity. - Smoking was not significantly associated with CA endpoints within the exposed group; residual confounding cannot be excluded. |
| [48] | - Carere et al.,   1995   - Italy | - 23 male nonsmoking PSAs; Rome. - 24 age-matched non-smoking controls. - Mean employment 22.4 years. | - CA and SCE in PBL. - High-frequency cells (HFC). - Personal benzene also measured. | Yes — personal benzene (mean 1.5 mg/m³; range 0.1–13.1) | Matched — non-smokers only | - CA: exposed 1.97% vs controls 1.46%; borderline significant (p=0.066). - SCE: exposed 4.73±0.15 vs controls 4.48±0.11 per cell (NOT significant; p=0.181). - HFC (75th percentile):   RR=1.3 (95% CI  1.1–1.5; significant).   - High benzene subgroup: CA RR=1.60 (95% CI   1.09–2.36); χ² trend p=0.0183 (significant).   - Blood lead: exposed   10.7 vs controls 8.4 µg/dL  (p=0.036). | - Mean personal benzene (1.5 mg/m³) was close to the cited ACGIH TLV-TWA of 1.6 mg/m³; the reported range shows that some individual measurements exceeded it. - CA increased across benzene-exposure categories and was significant in the high-exposure subgroup, supporting an exposure-response association rather than establishing a threshold. - Elevated blood lead indicates coexposure to leaded petrol additives (pre-unleaded fuel era). - SCE less sensitive than CA at these exposure levels. |

| **Ref** | **Author, Year Country** | **Population (M/F)** | **Biological Effect Assessed** | **Concurrent Exposure**  **Measured?** | **Smoking**  **Addressed?** | **Quantitative Results** | **Interpretation** |
| --- | --- | --- | --- | --- | --- | --- | --- |
| [49] | - Pitarque et al.,   1997   - Spain | - 42 male PSAs at 11 stations, Barcelona. - 45 male controls (university campus). - Personal benzene workers 0.91   mg/m³ vs  streets 0.06 mg/m³. | • SCE in PBL. • High-frequency cells (HFC). • Personal BTEX also measured. | Yes — personal BTEX (OVM  badge) | Matched | - SCE mean: exposed 7.99±0.27 vs controls 7.38±0.27 per cell. - HFC (95th percentile): 6.18±1.26% vs   4.38±1.06%  (p=0.02 by χ²-test).   - After statistical modelling controlling for lifestyle factors: occupational exposure NOT independently significant. - Cigarette smoking was the only significant predictor of SCE (p<0.001). - All benzene values below EU OEL (3.25 mg/m³) and ACGIH TLV (1.6 mg/m³) at time of study. | - Smoking confounds   SCE at these subOEL benzene concentrations — smoking control is essential.   - HFC elevated despite occupational exposure not being independently significant — sensitive endpoint. - Confirms importance of lifestyle factor adjustment in all PSA genotoxicity studies. |
| [53] | - Keretetse et al.,   2008   - South Africa | • 20 petrol attendants from  3 stations at  Potchefstroom.   - 20 unexposed controls. - Matched for age and smoking. | - Comet assay (alkaline; tail   intensity — DNA strand breaks) in PBL.   - Personal BTX also measured. | - Yes — personal BTX by GC-MS - Benzene mean 0.65 mg/m³; all below South African   OELs | Matched | - DNA damage   (comet, tail  intensity): exposed 15.06±9.10 vs unexposed 6.30±3.37 (p<0.05).   - After ANCOVA   adjusting for age and smoking: adjusted means 9.397 vs 7.638 (p<0.001).   - Calculated repair capacity: exposed 0.32±0.28 vs unexposed   0.25±0.24 (NOT significant).   - Volume of petrol sold correlated with benzene (r=0.634; p=0.004) and toluene (r=0.651; p=0.002). | - DNA strand breaks significantly   elevated despite all personal BTX below South African OELs.   - DNA damage was observed at   reported personal  BTX  concentrations below the South African OELs; the pilot study alone cannot determine the adequacy of the OELs.   - Volume of petrol sold is a significant   operational determinant of exposure — a practical metric for risk stratification.   - Calculated repair capacity did not differ significantly between groups; the study does not establish the mechanism of damage accumulation. |
| [67] | - Bindhya et al.,   2010   - India | • 27 petrol pump workers from  10 stations,  Coimbatore (23M, 4F). • 27 matched controls.   - Group I: <40 years; Group II: ≥40 years. - Exposure >8 years (n=10) and <8 years (n=17). | - CA (CTA and CSA) in PBL. - MN in lymphocytes   (CBMN;  cytochalasin-B).   - XRCC1 399 Arg/Gln polymorphism by PCR-RFLP. | No | Analysed by smoking status | - CTA — workers Group II (≥40 years): 2.94±1.24 vs Group II controls 1.19±0.83 (p<0.05). - CSA — workers Group II:   0.94±0.93 vs controls 0.38±0.62 (not reported as significant).   - MN — workers Group I (<40 years): 0.55±0.82 | - CTA was higher in older exposed workers than in older controls; the authors also reported higher cytogenetic damage with longer exposure, but the tabulated values do not support treating 16.13 and 5.85 as   CTA frequencies.   - Age and work duration were related in this sample, so their |

| **Ref** | **Author, Year Country** | **Population (M/F)** | **Biological Effect Assessed** | **Concurrent Exposure**  **Measured?** | **Smoking**  **Addressed?** | **Quantitative Results** | **Interpretation** |
| --- | --- | --- | --- | --- | --- | --- | --- |
|  |  |  |  |  |  | vs Group I controls 0 (p<0.05).   - Mean work duration was 16.13±4.56 years in worker Group II and   5.85±3.19 years in worker Group I; these are workduration values, not CTA frequencies.   - Smoking was associated with higher MN frequency in exposed vs control smokers (p<0.05); no significant association with CA was reported. - XRCC1 399 Arg/Gln polymorphism: no significant difference between workers and controls (OR=1; p=1.000). | independent contributions cannot be determined from the reported comparisons.   - MN was elevated in the younger worker group; the study did not establish agespecific genotoxic mechanisms. - XRCC1 polymorphism was not a significant susceptibility factor in this population. |
| [68] | - Sellappa et al.,   2010   - India | - 110 male petrol pump workers   from 21 stations,  Coimbatore.   - 100 matched controls. - Classified by smoking, alcohol, tobacco chewing habits. - Mean work duration: smokers 6.49±1.75 years; nonsmokers 5.83±0.92 years. | - Buccal cell MN assay (exfoliated cells; 3,000 cells per individual scored). - Binucleated cells (BN) and broken egg cells (BEC) also scored. | No | Yes —  stratified by smoking, alcohol and tobacco chewing | - MN per 3,000 cells (mean±SD): Nonsmokers: workers 11.17±1.06 vs controls 1.63±0.05 (p<0.001). - Smokers: workers 12.61±0.39 vs controls 2.79±0.16 (p<0.001). - Smokers with alcohol: workers 13.94±0.09 vs controls 3.64±0.02 (p<0.001). - Smokers with tobacco chewing:   workers  13.33±1.27 vs controls 3.73±0.13 (p<0.001).   - BN and BEC: significantly   higher in all worker groups vs corresponding controls (p<0.001 and p<0.01).   - Workers had approximately 7× higher MN than controls (nonsmokers group). | - MN was approximately sevenfold higher in non-smoking workers than in non-smoking controls, supporting an occupational association. - Higher MN frequencies were reported in lifestyle-factor   strata, but independent and additive effects of smoking, alcohol and tobacco chewing were not established by the reported comparisons.   - Largest buccal MN study in PSAs in this review (110 workers; 21 stations). - Absence of concurrent BTX measurement limits dose-response characterisation. |
| [28] | - Fracasso et al.,   2010   - Italy | - 28 service station attendants (SSA). - 33 petrochemical operators (PIO). - 21 pump maintenance workers   (GPMW).   - 51 non-exposed controls; all male. | - Comet assay (TM, TI, TL) in PBL; CA in PBL. - Personal benzene, urinary SPMA and ttMA also measured. | Yes — personal benzene by Radiello® passive sampler; urinary SPMA and ttMA | Yes — adjusted | - Personal benzene (median, µg/m³):   SSA: 40.00 (8.00– 260.00) vs controls 5.40 (p=0.0001).   - Comet TM: SSA 0.37±0.13 vs controls 0.28±0.08 (p=0.002). - Comet TI: SSA 2.78±0.92 vs controls 2.26±0.56 (p=0.023). - Comet TL: SSA   20.30±4.39 vs | - Three-domain study: personal   monitoring, urinary biomarkers and genotoxicity assessed in same subjects.   - Comet assay more sensitive than CA at sub-OEL benzene concentrations. - Personal benzene correlated with comet tail moment, |

| **Ref** | **Author, Year Country** | **Population (M/F)** | **Biological Effect Assessed** | **Concurrent Exposure**  **Measured?** | **Smoking**  **Addressed?** | **Quantitative Results** | **Interpretation** |
| --- | --- | --- | --- | --- | --- | --- | --- |
|  |  |  |  |  |  | controls 17.74±3.75 (p=0.002).   - CA (subgroup 19 SSA vs 16 controls): NOT significantly different. - Correlation personal benzene vs comet TM: SSA r=0.509 (p=0.007); GPMW r=0.525 (p=0.017); PIO r=0.420 (p=0.046). - SPMA median   (µg/g Cr): SSA 5.55 (1.55–15.00) vs controls 1.90 (p=0.0001). | supporting an exposure-effect association.  • SPMA was elevated and associated with benzene exposure, supporting its usefulness as a biomarker at these concentrations. |
| [5] | - Santiago et al.,   2014   - Brazil | - 60 GSAs (50M, 10F) from petrol stations in Rio de Janeiro. • No control group. - Median age 36 years; median exposure 16 years; 15% smokers. | • WCP FISH using 3colour probes for chromosomes 1, 2, and 4 (representing  21.87% of genome). • 100 metaphases per worker. | No | Analysed — 15% smokers; smoking not independently significant for  CA | - 10/60 workers   (16.6%) had high  CA frequency (>10 chromosomal abnormalities per 1,000 metaphases).   - 50/60 workers (83.4%) had no detectable aberrations above threshold. - Translocations: most frequent aberration (n=24); significantly more common than all other types (p=0.000). - Chromosome breakage: n=7;   deletions: n=13; monosomy: n=6; trisomy: n=1.   - Skin colour significantly associated with CA frequency   (p=0.002; OR=9.02; 95% CI 1.54–97.77).   - Female workers: higher proportion in high-CA group   (40%) vs males (12%); borderline  significant  (p=0.052;  OR=4.71).   - Age, duration of employment,   platelets,  leukocytes: NOT significantly associated with CA frequency. | - Translocations were the most frequent aberration observed, but the uncontrolled study cannot attribute them specifically to   benzene or determine their clinical significance.   - High-frequency CA was observed in 16.6% of workers; the study did not establish a clinical diagnostic threshold. - Skin colour was statistically associated with CA frequency, but the mechanism and the very wide confidence interval require cautious interpretation. - Female workers had a higher proportion in the high-CA group, but the association was borderline and based on a small subgroup. - No control group is a significant methodological limitation. |
| [54] | - Moro et al.,   2013   - Brazil | - 43 male GSAs (non-smokers). - 28 male controls. - Rio Grande do Sul. - Mean exposure   9.7±1.1 years. | - Comet assay (alkaline; DNA damage index) in PBL. • Buccal cell MN assay. - Personal BTEX and urinary ttMA also measured. | - Yes — personal BTEX by passive sampler - Benzene median 76.20 µg/m³ (range 54.34–   1285.48); all values below the cited | Non-smokers only | - DNA damage index (comet):   significantly higher in GSA vs controls.   - Buccal cell MN frequency: significantly higher in GSA vs controls. - Protein carbonyl and 3- | - Multi-domain study spanning personal air monitoring, biological monitoring and biological-effect assessment. - Benzene exposure values were below   the cited ACGIH TLV-TWA and were associated |

| **Ref** | **Author, Year Country** | **Population (M/F)** | **Biological Effect Assessed** | **Concurrent Exposure**  **Measured?** | **Smoking**  **Addressed?** | **Quantitative Results** | **Interpretation** |
| --- | --- | --- | --- | --- | --- | --- | --- |
|  |  |  |  | ACGIH TLVTWA of 1600 µg/m³ |  | nitrotyrosine: significantly higher in GSA.   - Antioxidant enzymes (SOD, CAT, GST) and   GSH: significantly lower in GSA.   - Duration of benzene exposure correlated with   DNA and protein damage.   - Specific comet and MN numerical values shown in figures only — not stated as text values. | with DNA damage and protein oxidation.   - Duration of exposure correlated with   DNA and protein damage, supporting a cumulative association without proving causality.   - Reduced antioxidant capacity (SOD, CAT, GST)   suggests oxidative stress mechanism mediates genotoxicity. |
| [34] | - Lovreglio et al.,   2014   - Italy | - 24 filling-station attendants. - 19 fuel-tanker drivers. - 31 controls; all male; Bari, Italy. | - CA and MN frequency in PBL. - Personal benzene and urinary biomarkers also measured. | - Yes — personal benzene by Radiello® passive sampler - Airborne benzene median 19.9 µg/m³ | Yes — adjusted | - CA: attendants 4.0±1.9% vs controls 3.7±1.9%   — NOT  significant.   - MN: attendants 8.0±3.0/1,000 BN vs controls 7.3±2.7   — NOT  significant.   - MN significantly dependent on airborne benzene in fuel-tanker   drivers (p=0.015) — NOT in  attendants.   - Urinary SPMA: attendants median 0.56 µg/g Cr vs controls 0.22 µg/g Cr (p<0.001). - Urinary benzene: attendants median 0.24 µg/L vs controls 0.12 µg/L (p<0.001). | - CA and MN NOT significant in filling-station attendants despite confirmed biological uptake (elevated SPMA and urinary benzene). - CA and MN did not differ significantly between attendants and controls at the reported exposure levels; these data do not establish a genotoxic threshold. - An MN-airborne benzene association was observed in the more highly exposed fuel-tanker drivers, but not in filling-station attendants. - In this study, urinary biomarkers distinguished exposed workers from controls when the cytogenetic endpoints did not. |
| [39] | - Martinez-   Valenzuela   - et al., 2017 - Mexico | - 60 GSAs; 60 controls. - Los Mochis, Sinaloa,   Mexico.   - Mean age exposed 37.9 vs controls 34.9 years (not significantly different). - 16 smokers (26.7%) and 44 non-smokers within exposed group. | - BMCyt assay in exfoliated buccal cells. - Endpoints: MN, pyknotic nuclei, chromatin condensation, nuclear buds, karyolytic cells, karyorrhexis, binucleated cells. - 3,000 cells per subject; Feulgen stain; coded slides. | No | Yes —  stratified by smoking and alcohol; FI calculated for all sub-groups | - MN: 6.6±0.5 vs 1.8±0.2 per 3,000 cells (p=0.001; FI=3.66). - Pyknotic nuclei:   78.4±9.6 vs 29.1±3.8 (p=0.001; FI=2.69).   - Chromatin condensation: 164.1±16.8 vs 11.3±1.5 (p=0.001; FI=14.52). - Nuclear buds:   6.3±0.8 vs 3.7±0.8  (p=0.030;  FI=1.70).   - Karyolytic cells:   4.8±0.6 vs 1.6±0.2 (p=0.000; FI=3.0).   - Karyorrhexis:   3.0±0.4 vs 1.5±0.2 (p=0.001; FI=2.0).   - Binucleated cells:   6.5±0.8 vs 2.6±0.7 | - Chromatin condensation shows the highest foldincrease (14.52) — the most discriminating endpoint in this study. - Nuclear abnormalities were higher in exposed workers, while smoking, alcohol use and years worked showed no   additional significant associations; the design does not prove gasoline vapour was the sole causal factor.   - No additional association with years worked was detected; this does |

| **Ref** | **Author, Year Country** | **Population (M/F)** | **Biological Effect Assessed** | **Concurrent Exposure**  **Measured?** | **Smoking**  **Addressed?** | **Quantitative Results** | **Interpretation** |
| --- | --- | --- | --- | --- | --- | --- | --- |
|  |  |  |  |  |  | (p=0.001;  FI=2.50).   - Non-smoking exposed vs nonsmoking controls: all biomarkers still significantly elevated (MN FI=4.17; chromatin condensation FI=12.38). - Smoking, alcohol consumption and   years worked had no statistically significant additional association with nuclearabnormality frequencies. | not establish when damage began or that it remained at a steady state.  • Largest BMCyt study in PSAs in this review. |
| [29] | - Pandey et al.,   2008   - India | • 100 petrol-pump workers from  15 stations, Lucknow; all male; ≥6 months exposure. • 100 matched controls. | - Comet assay (OTM, tail DNA%, tail length) in PBL. - MN in PBL. - Blood BTX by GCMS. - Air BTX by PID monitor. | Yes — air BTX (ppbRAE PID monitor) and  blood BTX by  GC-MS | Yes — matched | - Air benzene at petrol pump stations: 100–250 ppb; control sites: 5–10 ppb. - Blood benzene — PPWs: mean 7.94 ppb (n=39); controls: mean 2.82 ppb (p<0.01). - Comet assay: OTM 4.14±1.25 vs controls 3.29±0.92 (p<0.001); tail DNA% and tail length also significantly higher (p<0.001 and p<0.01). - MN frequency (subset n=39 PPWs, 18 controls): 14.79±3.92% vs 7.54±3.00% (p<0.001). - PPWs with high blood benzene had   significantly higher MN than PPWs with low blood benzene (p<0.001). | - Rare three-domain linkage: air BTX →   blood BTX →  genotoxic effect demonstrated in same subjects.   - Workers in the higher bloodbenzene category had higher MN frequency, supporting an internal-dose association. - The high-versus-low blood-benzene comparison supports an exposure-response relationship, although the observational design limits causal inference. - One of the most complete multidomain designs in the genotoxicity literature for PSAs. |
| [73] | - Salih et al., 2019 - Iraq | - 25 male petrol station workers. - 25 age-matched male controls. - Duhok city, Kurdistan. - Mean age   28.3±7 years; mean work  duration 4.1 years.   - All smokers and alcohol consumers excluded. | Buccal cell MN assay  (exfoliated cells; 1,000 cells per individual; Giemsa stain; Tolbert et al. criteria). | No | Matched — smokers and alcohol consumers excluded | - MN frequency per 1,000 cells:   workers mean 4.65  (reported dispersion ±2; range 0–14).   - Controls: mean 1.22 (range 0–6); the dispersion value is unclear in the published article. - Significantly higher in workers (p<0.05). - Duration of exposure significantly   correlated with  MN frequency (p<0.05): longer  duration = higher  MN. | - MN frequency approximately 3.8× higher in workers than controls after exclusion of lifestyle confounders. - Duration of exposure was associated with MN frequency, supporting a cumulative association. - Age was not significantly associated with MN frequency in this sample; this does not establish exposure duration as the only determinant. |

| **Ref** | **Author, Year Country** | **Population (M/F)** | **Biological Effect Assessed** | **Concurrent Exposure**  **Measured?** | **Smoking**  **Addressed?** | **Quantitative Results** | **Interpretation** |
| --- | --- | --- | --- | --- | --- | --- | --- |
|  |  |  |  |  |  | • Age: NOT  significantly  associated with MN frequency (p>0.05). | • Absence of concurrent BTX measurement limits dose characterisation. |
| [71] | - Metgud et al.,   2015   - India | - 40 male PSAs from 4 stations, Udaipur,   Rajasthan.   - 40 age-matched male controls. - Within each group: 20 smokers and 20 non-smokers. | - Buccal cell MN assay; nuclear anomalies: MN, binucleation, karyorrhexis, karyolysis. - 1,000 cells per slide;   Feulgen and  Giemsa stains. | No | Yes —  stratified by smoking status | - Feulgen stain — smokers of study vs control group: all four endpoints significantly higher in workers (p=0.00002). - Feulgen stain — non-smokers of study vs control group: significantly higher in workers (p=0.0034). - Giemsa stain — smokers of study vs control group: significantly higher in workers (p=0.0004). - Feulgen stain outperformed Giemsa for detecting nuclear anomalies. - Smoking significantly increased all nuclear anomalies in both exposed and control groups. - Specific mean frequency values per 1,000 cells not provided as text in   paper — significance statements only. | - Nuclear anomalies were higher in exposed workers in both smoking strata, supporting an occupational association. - Feulgen stain superior to Giemsa for nuclear anomaly detection in buccal cells — methodological recommendation for future studies. - Absence of specific numerical values in paper text limits precision of comparison with other studies. |
| [40] | - Shaikh et al.,   2021   - India | - 70 male PSAs from 10 petrol pumps in heavy-traffic areas,   Ahmedabad.   - 15–20 years exposure; 8–12 h/day; ages 30– 46. - 70 age-matched male controls from lowpollution corporate areas. | - CBMN assay (MN in binucleated cells; ≥1,000 BN cells per subject; Giemsa). - SCE assay (BrdU incorporation;   Hoechst  33258/UV/Giemsa;  100 M2 metaphases per subject).   - CCPI, AGT, PDT calculated. - In vitro vitamin C (20 mM) and vitamin E (10 mM) amelioration study. | No | Non-smokers only | - MN (CBMN; mean±SE per 1,000 BN cells):   Exposed:  17.39±0.58 vs Controls: 6.09±0.40  (p<0.001).   - Exposed + vitamin   C: 14.72±0.65 (significantly reduced; p<0.001).   - Exposed + vitamin   E: 14.33±0.65 (significantly reduced; p<0.001).   - SCE (mean±SE): Exposed:   85.63±2.15 vs Controls: 75.80±1.85 (p<0.001).   - CCPI (mean±SE): Exposed:   1.93±0.03 vs Controls:  2.33±0.01 (p<0.001;  significantly lower — reduced  proliferative capacity).   - AGT (h): Exposed   37.85±0.67 vs | - Reduced CCPI and prolonged   AGT/PDT indicate  altered cell proliferative capacity; the study did not establish a pre-neoplastic clinical state.   - In vitro treatment with vitamins C and E significantly but incompletely   attenuated the measured genotoxicity endpoints; this does not demonstrate clinical benefit from worker supplementation.   - Comprehensive multi-endpoint genotoxicity assessment (MN, SCE, CCPI, AGT, PDT) — most thorough genotoxicity study reviewed. - Health complaints: headache 15%; body ache 15%; allergy 20%; |

| **Ref** | **Author, Year Country** | **Population (M/F)** | **Biological Effect Assessed** | **Concurrent Exposure**  **Measured?** | **Smoking**  **Addressed?** | **Quantitative Results** | **Interpretation** |
| --- | --- | --- | --- | --- | --- | --- | --- |
|  |  |  |  |  |  | Controls 30.85±0.21 (p<0.001).   - PDT (h): Exposed   12.51±0.22 vs  Controls 10.28±0.07 (p<0.001).   - In vitro vitamin C and vitamin E treatment significantly improved all measured biomarkers but did not fully restore them to control levels. | breathing problems 11%. |
| **GROUP B — HAEMATOLOGICAL AND BIOCHEMICAL EFFECTS** | | | | | | | |
| [65] | - Jakobsson et al.,   1993   - Sweden | - Male petrol station attendants in Sweden. - Historical cohort based on the 1970 Swedish census; cancer follow-up   1971–1984. | Acute myeloid leukaemia (AML) incidence in retrospective cohort; registry-based cancer follow-up. | No — exposure reconstructed from job history | Not applicable  —  epidemiological cohort study | - Ten AML cases were observed among male petrol station attendants versus 2.8 expected   (observed/expected ratio 3.6; 95% CI 1.7–6.6).   - Reconstructed work histories of the 10 cases were compatible with petrol and benzene exposure; the authors noted that petrol-station airborne benzene levels were lower than levels previously associated with leukaemia. - The authors considered that benzene, dermal uptake, or other petrol/exhaust components might have contributed to the excess risk. | - Early epidemiological study reporting an association between   petrol-station employment and AML.   - The registry-based design allowed assessment of a malignancy with a potentially long latency period. - The excess supports an occupational association, but the study could not isolate benzene from dermal exposure or other petrol and exhaust components. |
| [77] | - Rushton & - Romaniuk, 1997 - UK | - Male petroleum marketing and distribution workers in the   UK.   - Nested casecontrol within occupational cohort;   multiple employment periods. | Leukaemia incidence (all subtypes); doseresponse analysis vs quantitative benzene exposure reconstructed from historical monitoring data. | No —  retrospective exposure reconstruction | Not applicable | - No significant increase in overall leukaemia risk with higher cumulative benzene exposure or exposure intensity; risk was consistently about doubled among workers employed for >10 years. - For acute myeloid and monocytic leukaemia, cumulative exposure of 4.5–45 ppm-years versus <0.45 ppm-years gave OR=2.8 (95% CI 0.8–9.4). - Mean intensity of 0.2–0.4 ppm versus <0.02 ppm gave OR=2.8 (95% CI 0.9–8.5); neither estimate | - The study found no evidence of an association between benzene exposure and acute or chronic lymphoid leukaemia. - Results suggested a possible relation with acute myeloid and monocytic leukaemia, but confidence intervals were wide and exposureresponse findings were not consistent across analyses. - Uncertain work histories, exposure estimates and small exposure subgroups limit causal interpretation. |

| **Ref** | **Author, Year Country** | **Population (M/F)** | **Biological Effect Assessed** | **Concurrent Exposure**  **Measured?** | **Smoking**  **Addressed?** | **Quantitative Results** | **Interpretation** |
| --- | --- | --- | --- | --- | --- | --- | --- |
|  |  |  |  |  |  | was statistically significant.  • Peaked exposures appeared more common in acute myeloid/monocytic cases, but small numbers prevented separation of peaked from unpeaked exposure effects. |  |
| [41] | - Schnatter et al.,   2012   - International - (Australia,   Canada, UK) | - Pooled analysis: 370 LH cancer case subjects + 1,587 matched controls. - Petroleum distribution workers; all male. - Canada (1964– 1994); UK   (1950–2005);  Australia  (1981–2006). | - LH cancer subtypes:   AML (n=60), CLL  (n=80), CML (n=28), MDS (n=29), MPD (n=30).   - Quantitative benzene exposure in ppm reconstructed; conditional logistic regression; doseresponse analysis. | - No —   retrospective quantitative exposure reconstruction  from  historical monitoring data   - Mean exposures 0.2–0.3 ppm; average cumulative <10 ppmyears | Data largely missing (UK study); sensitivity analyses performed | - MDS — monotonic dose-response with cumulative benzene (p trend=0.01): Lowest tertile (≤0.348 ppm-   years): referent.   - Middle tertile (0.348–2.93 ppmyears): OR=1.73 (95% CI 0.55– 5.47). - Highest tertile (>2.93 ppm-years):   OR=4.33 (95% CI 1.31–14.3; p global=0.03).   - Peak exposure >3 ppm for MDS in   high/medium certainty diagnoses: OR=6.32 (95% CI 1.32–30.2).   - Median cumulative benzene — MDS cases: 3.4 ppmyears vs controls 1.4 ppm-years. - AML: no   statistically significant doseresponse (p global=0.62);  highest tertile  OR=1.39 (95% CI 0.68–2.85).   - CLL, CML, MPD:   no consistent doseresponse relationships. | - MDS showed the clearest exposureresponse association at the benzene exposures assessed; AML, CLL, CML and MPD did not show consistent doseresponse relationships. - Diagnostic reclassification may partly explain differences from earlier AML findings. - OR=4.33 in the highest cumulativeexposure tertile supports an association between benzene exposure   and MDS but does not by itself establish causality.   - Largest international pooled analysis of benzene-LH cancer associations in petroleum workers. |
| [42] | - Udonwa et al.,   2009   - Nigeria | - 50 petrol station attendants (PSA). - 50 automobile mechanics (AM). - 50 general population controls. - Calabar, Nigeria; all ≥18 years. - 100% of PSAs: no PPE, no face masks, no rubber-hooded pumps. | • MetHb (%) by spectrophotometry (Evelyn and  Malloy; 630 nm). • PCV (%) by microhaematocrit. | No | Not addressed | - Mean MetHb: AM 7.7% vs PSA 5.8% vs controls 2.7% (all significantly higher than controls; p<0.05). - Mean PCV: PSA   30.8% vs AM  33.3% vs controls 40.8% (PSA  significantly lower than controls; p<0.05).   - MetHb in AM significantly higher than PSA (p<0.05) —   attributed to additional dermal/ingestion routes.   - Duration-response   (PSA MetHb): <1 | - MetHb increased and PCV decreased across self-reported exposure-duration groups; workers with >10 years had mean MetHb 13.6% and PCV   28.0%.   - These findings are consistent with impaired oxygencarrying capacity and low packed cell volume, but the cross-sectional study did not establish a clinical diagnosis in individual workers. - The reported 6–8% benzene content of Nigerian PMS may |

| **Ref** | **Author, Year Country** | **Population (M/F)** | **Biological Effect Assessed** | **Concurrent Exposure**  **Measured?** | **Smoking**  **Addressed?** | **Quantitative Results** | **Interpretation** |
| --- | --- | --- | --- | --- | --- | --- | --- |
|  |  |  |  |  |  | year 1.8%; 1–2 years 2.5%; 3–4 years 7.2%; 5–6 years 7.8%; >10 years 13.6%.   - PCV inversely proportional to duration: <1 year   40.4%; progressively decreasing to 28.0% at >10 years.   - 8% of PSAs admitted entering underground fuel storage to measure volume (extremely hazardous). - The article reported that Nigerian PMS contained 6–8% benzene, compared with approximately 1% in industrialized countries. | have contributed to exposure, although benzene exposure was not directly measured in participants.  • Lack of PPE and reported entry into  underground  storage facilities indicate important occupational-health risks. |
| [43] | - Nwanjo & Ojiako, - 2007 - Nigeria | - 20 petrol station attendants, Owerri Metropolis, Imo State. - 20 healthy controls (no petrol exposure). - Ages 16–38 years; divided by exposure duration: 1–5 years and 6–10 years. | - Liver enzymes (AST, ALT, ALP) by standard colorimetric methods. - Kidney function:   creatinine, urea by Jaffe's and colorimetric methods.   - Urinalysis for proteinuria. - Total, conjugated and unconjugated bilirubin. | No | Not addressed | - Kidney function — 6–10 years exposure vs controls: Creatinine:   1.6±0.36 vs 0.98±0.21 mg/dL (p<0.05).   - Urea: 50.4±1.90 vs 34.6±5.04 mg/dL (p<0.05). - Proteinuria: trace in 6–10 years group; nil in controls and 1–5 years group (p<0.05). - Liver function — 6– 10 years exposure vs controls: AST: 56.82±3.64 vs   32.26±2.68 IU/L (p<0.05).   - ALT: 48.2±3.35 vs 30.8±2.9 IU/L (p<0.05). - ALP: 110.2±8.2 vs 86.01±4.61 IU/L (p<0.05). - All bilirubin fractions: NOT significantly different at any exposure duration. - 1–5 years exposure group: NO significant differences from controls for any parameter. | - Liver enzymes and renal indices differed from controls in the 6– 10-year group but not in the 1–5-year group; the twogroup comparison does not establish a toxicity threshold. - The findings support attention to duration of employment, but the study did not determine when occupational surveillance should begin. - Trace urinary protein together with higher creatinine and urea may indicate renal involvement, but clinical renal injury   was not independently confirmed.   - Absence of BTX measurement limits attribution to a specific petrol constituent. |
| [74] | - Ajugwo et al.,   2014   - Nigeria | - 35 fuel attendants (17M, 18F); Elele, Rivers State. - 35 automobile mechanics (all male). - 30 healthy control | Full haematological panel: RBC, WBC, PCV, Hb, MCV, MCH, MCHC.  Manual methods; SPSS v14. | No | Not addressed | - Haematological indices — fuel attendants vs controls   (mean±SD): RBC  (×10¹²/L):  4.88±0.67 vs 5.42±0.71 (p<0.05).   - Hb (g/dL):   12.50±1.33 vs | • The authors reported lower haematological indices in fuel attendants and auto mechanics, with fuel attendants considered more at risk; the comparisons do not isolate direct |

| **Ref** | **Author, Year Country** | **Population (M/F)** | **Biological Effect Assessed** | **Concurrent Exposure**  **Measured?** | **Smoking**  **Addressed?** | **Quantitative Results** | **Interpretation** |
| --- | --- | --- | --- | --- | --- | --- | --- |
|  |  | students; ages 15–30 years.  • Fuel attendants: T1 (≤2 years) and T2 (>2 years). |  |  |  | 13.55±1.49 (p<0.05).   - MCH (pg):   24.67±2.64 vs 25.99±2.24 (p<0.05).   - MCHC (g/dL):   31.43±1.96 vs  31.74±0.98 (p<0.05).   - WBC and PCV: NOT significantly different from controls. - The authors reported lower RBC, Hb, MCH and MCHC in fuel attendants and auto mechanics than in controls (p<0.05). - Duration effect: attendants exposed for >2 years had   lower PCV, Hb, MCH and MCHC  than attendants exposed for ≤2 years (p<0.05).   - NOTE: 18/35 fuel attendants were female — highest female PSA proportion of any reviewed Nigerian study. | dispensing as the cause.   - Lower PCV, Hb, MCH and MCHC   in attendants employed for >2 years support an  association with  employment  duration, not a proven onset time.   - The inclusion of 18 female attendants provides   uncommon  haematological data for female PSAs in this setting.   - The pattern is compatible with anaemia risk, but benzene exposure and bone-marrow suppression were not directly measured. |
| [44] | - Abou-ElWafa et al., - 2015 - Egypt | - 102 male PSAs from 6 stations, Mansoura city. - 102 male office/service worker controls. - All male; no PPE; no preemployment or periodic examination. - Median employment duration 10 years (range 0.1–40). | Full blood count; liver enzymes (ALT, AST); kidney function (urea, uric acid, creatinine); serum albumin; total protein. | No | Yes — smoking duration longer in PSAs (p=0.02); included as variable | - Haematological findings   (mean±SD): RBC  (million cells/mcL): PSA 4.8±0.4 vs controls 5.3±0.5 (t=7.9; p≤0.001).   - Hb (g/dL): PSA   13.9±1.3 vs     - HCT (%): PSA   39.7±3.4 vs     - Hb <12 g/dL: 7   (6.9%) PSAs vs 1  (0.98%) control (p=0.06; not statistically significant).   - Platelets, WBC,   MCV, MCH, MCHC: NOT significantly different.   - Biochemical findings: ALT (median IU/L): PSA 30 (12–121) vs controls 26 (11– 125) (Z=2.8; p=0.004;   SIGNIFICANT).   - AST, uric acid, creatinine, urea, serum albumin, total protein: NOT significantly different. | - RBC, Hb and HCT were significantly lower in the 102 Egyptian PSAs than   in controls, supporting an occupational haematological association.   - Hb <12 g/dL was more frequent in PSAs (6.9% vs 0.98%), but this comparison was not statistically significant (p=0.06). - Median ALT was higher, but the proportion above 45 U/L was   identical in both groups and AST was not different; the clinical significance of the isolated median difference is uncertain.   - This study included 102 Egyptian PSAs and 102 controls. |

| **Ref** | **Author, Year Country** | **Population (M/F)** | **Biological Effect Assessed** | **Concurrent Exposure**  **Measured?** | **Smoking**  **Addressed?** | **Quantitative Results** | **Interpretation** |
| --- | --- | --- | --- | --- | --- | --- | --- |
|  |  |  |  |  |  | • Only ALT  significantly elevated; all renal parameters within normal range. |  |
| [69] | - Mahmood, 2012 - Iraq | • 48 gasoline filling workers;  27 agematched controls.   - Sulaimani city; ages 27–65 years (mean 45.0±10.8). - Mean exposure 20.2±11.6 years. | - Liver enzymes   (GOT/AST, GPT/ALT,  bilirubin) by autoanalyser.   - Full blood count by haematometer (Coulter Swelab). - Trace elements (Cu, Zn, Fe) by ICP atomic absorption spectrophotometry. | No | Not stated | - Liver transaminases (mean±SD):   GOT/AST:  workers 43.1±12.9 vs controls  29.8±10.9 IU/L (p<0.05; +44.6%).   - GPT/ALT: workers 39.5±22.2 vs controls 24.1±12.2 IU/L (p<0.05; +64%). - Total bilirubin:   workers 0.80±0.2 vs controls  0.88±0.3 mg/dL (NOT significant).   - Haematology: platelets significantly decreased in workers (−13%; p<0.05); other parameters not significantly affected. - Trace elements: Serum Cu: workers significantly elevated (+15%; p=0.01). - Serum Zn: workers significantly elevated (+15.6%; p=0.003). - Serum Fe: workers significantly decreased (−29.3%; p=0.0001). - Cu/Zn ratio: NOT significantly different between groups. - No correlation between trace elements and duration of exposure. | - ALT and AST were higher in workers after a mean 20.2 years of employment, supporting a liverenzyme association without   establishing clinical hepatocellular injury.   - Platelet count was 13% lower in workers; benzene exposure and bonemarrow suppression were not directly measured. - Lower serum iron and higher copper and zinc indicate altered traceelement levels, but the study did not establish oxidativestress-related redistribution as the mechanism. - Absence of BTX measurement limits attribution to specific compounds. |
| [31] | - Neghab et al.,   2015   - Iran | - 200 PSA employees from all 20 private petrol stations in Shiraz (194M, 6F). - 200 unexposed controls. - Rotating 8-hour shifts. | - Liver function tests (AST, ALT, ALP, serum albumin, total protein, bilirubin). - Kidney function (urea, creatinine, calcium, sodium, potassium). • Personal BTX also measured. | - Yes — personal BTX by GC-FID - Benzene GM 0.8 mg/m³; toluene GM 1.4 mg/m³; xylene GM   2.8 mg/m³; all below ACGIH  TLVs | Yes —  included in multiple regression | - Liver function (exposed vs controls; mean±SD): ALT:   23.1±13.9 vs 19.4±10.4 U/L (p=0.018).   - AST: 24.14±9.2 vs 22.3±8.7 U/L (p=0.04). - Direct bilirubin:   0.19±0.14 vs 0.15±0.06 mg/dL (p<0.001).   - Serum albumin:   4.5±0.5 vs 4.8±0.4 g/dL (p<0.001; lower in exposed).   - Total protein:   7.36±0.8 vs  8.2±0.7 g/dL | - This study combined personal BTX measurement with liver- and kidneyfunction markers in the same worker population. - Subtle, subclinical differences in liver- and kidneyfunction parameters were associated   with measured BTX  concentrations below the cited TLVs; the crosssectional study does not determine whether current TLVs are protective. |

| **Ref** | **Author, Year Country** | **Population (M/F)** | **Biological Effect Assessed** | **Concurrent Exposure**  **Measured?** | **Smoking**  **Addressed?** | **Quantitative Results** | **Interpretation** |
| --- | --- | --- | --- | --- | --- | --- | --- |
|  |  |  |  |  |  | (p<0.001; lower in exposed).   - ALP: NOT   significant (p=0.09).   - Kidney function: Urea: 36±5.4 vs   34±8.2 mg/dL  (p<0.001);  Creatinine: 1.1±0.3 vs 1.05±0.14 mg/dL (p=0.05).   - Calcium: 9.2±0.7 vs 9.7±0.6 mg/dL   (p<0.001; LOWER in exposed).   - Sodium: 139±2.6 vs   140±2.6 mEq/L  (p=0.008; LOWER in exposed).   - Associations persisted after adjustment for age, smoking and BMI. | - Lower albumin and total protein may reflect altered liverfunction parameters, but clinical hepatic synthetic dysfunction was not established. - The exposed group included 6 female PSAs (3%); sexspecific results were not reported. |
| [70] | - Beerappa et al.,   2013   - India | - 79 petrol fillers (attendants) from stations in Bangalore. - 21 cashiers at same stations (internal control group). - Ages 20–41 years; 79% non-smokers. | - Urinary 8-oxodG (8oxo-7,8-dihydro-   2'-  deoxyguanosine) by competitive ELISA (Japan  Institute for the Control of Ageing kit).   - Creatinine by Jaffe's method. - Spearman rank correlation; median test. | No — volume of petrol sold used as proxy for exposure | Yes —  stratified by smoking; alcohol; tobacco chewing; food habits | - 8-oxodG levels (µg/g creatinine): Petrol fillers median: 1.86 µg/g Cr. - Cashiers median:   0.47 µg/g Cr.   - Significantly higher in petrol fillers than cashiers (p=0.001; median test). - By age group (total population; mean±SE): ≤20 years 4.65±2.00; 21–30 years   2.97±0.56; 31–40 years 1.57±0.32;  ≥41 years  4.08±1.99 µg/g Cr.   - Age group differences NOT significant (p>0.05). - Significant positive Spearman   correlation between working hours per day and 8-oxodG in both petrol fillers and cashiers. | - Petrol fillers had approximately   fourfold higher  median 8-oxodG  than cashiers at the same stations, supporting an association with filling work; the exposure route was not directly measured.   - Working hours per day correlated with   8-oxodG, supporting an association with daily work duration.   - Age-group differences were not statistically significant; this does not establish that working hours outweigh age as a predictor. - Comparing fillers with cashiers at the same stations may reduce some station-level differences, although residual confounding remains possible. |
| **GROUP C — OXIDATIVE STRESS AND IMMUNOLOGICAL EFFECTS** | | | | | | | |
| [23] | - Xia et al., 2017 - China | - 96 petrol fillers at refuelling areas; 48 cashiers in offices. - 9 filling stations,   Nanning,  Guangxi.   - Primary focus:   Mn (MMT  additive) coexposure. | - Oxidative stress panel: SOD, GSHPx, CAT, MDA (plasma); Hsp70 (plasma, ELISA). - BTEX and Mn TWA at refuelling vs office areas also measured. | - Yes — BTEX and Mn TWA   at refuelling vs office areas   - All BTEX below Chinese national occupational standards | Current smoking was recorded (16.7% exposed; 20.8% controls) but was not adjusted for in the reported group comparisons. | - Oxidative stress (mean±SD; exposed vs controls): SOD: 39.18±6.05 vs   52.84±3.87 U/mL  (p<0.001; LOWER in exposed).   - GSH-Px:   186.07±15.63 vs  194.38±10.42 U/mL (p=0.001; lower in exposed).   - MDA: 1.68±0.52 vs   1.43±0.64 nmol/L | - Oxidative-stress markers differed between fillingstation operators and office workers although measured BTEX and Mn   concentrations were below the cited Chinese occupational standards.   - Associations of BTEX cumulative exposure index |

| **Ref** | **Author, Year Country** | **Population (M/F)** | **Biological Effect Assessed** | **Concurrent Exposure**  **Measured?** | **Smoking**  **Addressed?** | **Quantitative Results** | **Interpretation** |
| --- | --- | --- | --- | --- | --- | --- | --- |
|  |  |  |  |  |  | (p=0.021; HIGHER in exposed).   - Hsp70: 2.77±0.64 vs 2.32±0.87 ng/mL (p=0.002; HIGHER in exposed). - CAT: 3.02±0.95 vs   2.91±0.76 U/mL  (NOT significant; p=0.494).   - BTEX CEI inversely correlated with SOD (r=−0.508; p<0.001) and   positively with MDA (r=0.367; p<0.001).   - Mn TWA in   refuelling areas:  6.44–127.34 µg/m³ vs offices 3.16– 7.22 µg/m³ (p<0.05).   - Hsp70 correlated with BTEX CEI (r=0.328; p<0.01) and Mn CEI (r=0.437; p<0.001). | with SOD and MDA support an exposure-response relationship but do not establish a direct causal mechanism.   - Higher Hsp70 and its association with   BTEX and Mn cumulative exposure indices support its potential as a cellular-stress biomarker in this setting.   - Concurrent BTEX and Mn exposure prevents the independent contribution of either exposure from being determined from these results. |
| [55] | - Moro et al.,   2015   - Brazil | - 60 male GSAs (44 nonsmokers, 16 smokers). • 28 non-smoking controls. - Rio Grande do Sul; mean exposure ~10 years. | - Immunological panel: CD80 and CD86 expression on monocytes (flow cytometry). - Serum IL-8 (ELISA). - ALA-D enzyme activity. - Personal BTEX and urinary ttMA also measured. | - Yes — personal BTEX by passive sampler - Benzene median 144.2   µg/m³; all below ACGIH limits | Analysed by smoking status | - CD80 expression on monocytes: significantly decreased in GSA vs controls (p<0.05). - CD86 expression: significantly decreased in GSA vs controls (p<0.05). - IL-8: significantly elevated in GSA vs controls (p<0.05). - ALA-D activity: significantly reduced in GSA vs controls. - Multiple regression: urinary ttMA only factor significantly contributing to   CD80 reduction (β=−0.278; p=0.044) and CD86 reduction (β=−0.379; p<0.001).   - Exposure time was the only significant factor in the ALAD regression model (β=0.752; p=0.001); the authors reported longer exposure as   associated with lower ALA-D activity.   - Urinary ttMA (median, IQR; µg/g Cr): GSA non-smokers 320.0 (190.0–450.0); smokers 400.0 (300.0–600.0); | - Lower monocyte CD80/CD86   expression and higher IL-8 indicate  immunological alterations; functional antigenpresentation capacity or clinical immunosuppression was not directly assessed.   - Urinary ttMA was independently associated with lower monocyte CD80 and CD86 expression, supporting an association with benzene exposure. - ALA-D activity was lower and associated with   exposure duration and BTX  biomarkers; lead was not detected in the analysed gasoline samples, so the finding should not be attributed to lead exposure.   - The study combined personal BTX monitoring, urinary exposure biomarkers and biological-effect measurements. |

| **Ref** | **Author, Year Country** | **Population (M/F)** | **Biological Effect Assessed** | **Concurrent Exposure**  **Measured?** | **Smoking**  **Addressed?** | **Quantitative Results** | **Interpretation** |
| --- | --- | --- | --- | --- | --- | --- | --- |
|  |  |  |  |  |  | controls 70.0 (50.0–120.0; p<0.001). |  |
| [45] | - Toson et al.,   2022   - Egypt | - 62 male GSAs (ages 16–70 years; ≥8 h daily). - 29 age- and sexmatched controls. - Damietta governorate. | - TNF-α (ELISA);   AMCase  (colorimetric).   - TNF-α/AMCase and NO/AMCase   ratios; NLR and PLR.   - SOD; catalase; GSH; MDA; NO; TAC. - CBC (Mindray BC2600). | No | Not addressed | - TNF-α (ng/100 mL): 186.7±20 vs 134±19 (p<0.05). - AMCase (U/L):   20.12±1.3 vs 19.89±0.84 (NOT significant; p>0.05).   - TNF-α/AMCase   ratio: 9.5±0.6 vs 5.44±1.14 (p<0.05).   - NLR: 155.8±8.01 vs   187.2±12.4  (p<0.05; LOWER in exposed).   - PLR: 98.65±3.98 vs 128.3±6.4   (p<0.001; LOWER in exposed).   - Lymphocytes:   284±9 vs 207±8  ×10⁵/µL (p<0.001; HIGHER in exposed).   - Catalase (U/L):   372.5±30.9 vs  481.1±43.5  (p<0.01;  LOWER).   - GSH (mg/dL):   22.76±1.6 vs  30.76±8.6  (p<0.001;  LOWER).   - MDA (µmol/mL packed cells ×10⁻⁶):   7.794±0.613 vs  6.453±0.067 (p<0.05;  HIGHER).   - SOD and TAC: NOT significantly different. | - Higher TNF-α and TNF-α/AMCase   ratio were interpreted by the authors as supporting M1  macrophage  polarization, but macrophage phenotype was not measured directly.   - TNF-α/AMCase was proposed as an indicator of relative M1/M2 dominance; further validation is required. - Higher lymphocyte count with lower NLR and PLR   indicates altered circulating inflammatory-cell indices in exposed workers.   - Lower catalase and GSH with higher MDA is consistent with oxidative stress, but the cross-sectional findings do not establish it as the mechanistic driver of the immunological changes. - SOD and total antioxidant capacity did not differ significantly, indicating that not all antioxidant measures changed in the same direction. |
| [46] | - Ahmadi et al.,   2019   - Iran | - 40 male GSAs; 39 male controls. - Kerman city; all non-smokers. - Work experience >1 year; 40 h/week. | - Hb, HCT, RBC by Sysmex KX-21. - Oxidised Hb, MetHb, haemichrome by spectrophotometry (NanoDrop 2000; 560, 577, 630 nm). - SAC by FRAP test. - Protein oxidation (protein carbonyl) by spectrophotometry. | No | Non-smokers only | - Hb (g/dL): exposed 16.76±0.14 vs controls 15.25±0.14   (p<0.001; HIGHER in exposed — paradoxical; attributed to compensatory secondary polycythaemia).   - HCT (%):   49.11±0.36 vs  45.37±0.31  (p<0.001; HIGHER in exposed).   - RBC (mil/µL):   5.85±0.06 vs  5.33±0.06 (p<0.001; HIGHER in exposed).   - MetHb (g/dL):   1.07±0.07 vs  0.39±0.04  (p<0.001; | - Hb, HCT and RBC were higher in exposed workers; the authors proposed a response similar to secondary polycythaemia, but the cross-sectional study did not establish a compensatory mechanism. - MetHb was higher and oxyhaemoglobin was lower in exposed workers,   indicating altered haemoglobin oxidation and potentially reduced effective oxygencarrying haemoglobin.   - Higher haemichrome provides evidence of oxidised haemoglobin |

| **Ref** | **Author, Year Country** | **Population (M/F)** | **Biological Effect Assessed** | **Concurrent Exposure**  **Measured?** | **Smoking**  **Addressed?** | **Quantitative Results** | **Interpretation** |
| --- | --- | --- | --- | --- | --- | --- | --- |
|  |  |  |  |  |  | significantly  HIGHER).   - Haemichrome (g/dL): 0.80±0.07 vs 0.37±0.02 (p<0.001;   significantly  HIGHER).   - FRAP (mM; antioxidant capacity): 0.23±0.01 vs 0.34±0.01 (p<0.001;   significantly  LOWER).   - Protein carbonyl (mmol/mg   protein): 7.47±0.33 vs 5.81±0.19 (p<0.001;  significantly  HIGHER). | products; the study did not establish the clinical severity of this change.   - Lower FRAP antioxidant capacity and higher protein carbonyl are consistent with increased oxidative stress. - The authors reported no previous study of haemichrome formation in gasoline-exposed workers, making it a potentially useful marker for further investigation. |
| **GROUP D — PULMONARY FUNCTION** | | | | | | | |
| [9] | - Singhal et al.,   2007   - India | - 30 healthy nonsmoking male   PPWs  (working >1 year; ≥10 h/day; ages 20– 40).   - 30 age-matched healthy nonsmoking male hospital controls. - Delhi. | - Spirometry: FVC, FEV₁, FEV₁/FVC,   FEF₂₅₋₇₅, PEFR, PIFR, FIF₅₀, FIVC, IC, ERV.   - Computerised spirometer (Spiro 232; P.K. Morgan); ATS guidelines; best of 3 manoeuvres. | No — no  concurrent individual BTX  measurement;  the article cited  CPCB ambient  data for the  vicinity of petrol  pumps (SPM 301±71 µg/m³; RSPM 135±46  µg/m³), both above the cited limits. | Non-smokers only | - Flow rates (mean±SD; workers vs controls): PEFR (L/min): 5.41±1.79 vs 7.25±1.55 (p<0.05). - FEF₂₅₋₇₅ (L/min):   3.48±1.62 vs 4.83±1.27 (p<0.05).   - PIFR (L/min):   2.17±0.58 vs  3.52±1.10 (p<0.05).   - FIF₅₀ (L/min):   1.86±0.61 vs 3.24±1.10 (p<0.05).   - Lung volumes   (mean±SD): FVC  (L): 3.00±0.75 vs 3.96±0.34 (p<0.05).   - FEV₁ (L): 2.65±0.67 vs 3.57±0.41 (p<0.05). - FEV₁/FVC (%):   89.25±13.34 vs 90.31±9.02 (NOT significant).   - ERV (L): 0.61±0.51 vs 1.24±0.42 (p<0.05). - FIVC and IC: NOT significantly different. | - Lower FVC and FEV₁ with no significant   difference in FEV₁/FVC is consistent with the restrictive pattern described by the authors.   - Lower FEF₂₅₋₇₅ and FIF₅₀ suggests small-airway involvement, although these indices can be reduced in both restrictive and obstructive disease. - No concurrent BTX measurement was performed, and workers were also exposed to trafficrelated particulate and gaseous pollutants; the responsible agent cannot be identified. - FVC was 0.96 L lower in workers than controls and statistically significant; the study did not independently assess the clinical significance of this difference. |
| [7] | - Madhuri & - Chandrashekar,   2012   - India | - 30 healthy nonsmoking male petrol pump workers (working >3 years). - 30 healthy nonsmoking male hospital staff controls. - Kanchipuram,   Tamil Nadu. | - Spirometry: FVC, FEV₁,   FEV₁/FVC%,  FEF₂₅₋₇₅, PEFR.   - Computerised spirometer (Helios 401 RMS); tests repeated three times and the best matching result analysed. | No | Non-smokers only | - FVC (L): workers 1.97±0.348 vs controls   2.24±0.44; FEV₁  (L): 1.68±0.286 vs 2.15±0.39 (both p<0.01).   - FEF₂₅₋₇₅: workers 0.86±0.074 vs controls   0.96±0.05; PEFR:  4.30±0.85 vs  5.05±0.70 (both p<0.01). | - Lower FVC and FEV₁ with no significant   FEV₁/FVC  difference is consistent with the restrictive pattern described by the authors.   - The authors stated that exposure beyond three years changed the pattern to mixed |

| **Ref** | **Author, Year Country** | **Population (M/F)** | **Biological Effect Assessed** | **Concurrent Exposure**  **Measured?** | **Smoking**  **Addressed?** | **Quantitative Results** | **Interpretation** |
| --- | --- | --- | --- | --- | --- | --- | --- |
|  |  |  |  |  |  | - FEV₁/FVC: no significant difference (p>0.05). The article reports values of 3.68±1.018 vs 3.85±0.76, but the unit or scale is unclear and is inconsistent with a conventional FEV₁/FVC ratio. - The authors stated that workers with longer employment had lower percentages of predicted values for most parameters, but duration-specific numerical results and statistical tests were not provided. | impairment; however, all workers had >3  years of employment and no duration subgroups or diagnostic obstruction criterion were reported to substantiate progression.   - A possible duration relationship was described, but the absence of duration-specific data and statistical testing limits that conclusion. - No personal or environmental exposure measurements were reported, limiting attribution to petrol vapour or a specific constituent. |
| [6] | - Solanki et al.,   2015   - India | - 227 nonsmoking petrol pump workers from 53 stations in 5 zones of   Ahmedabad (working ≥1 year; ≥8 h/day; ages 20–50).   - 227 age-, sex-, height-, weightmatched nonsmoking office worker controls. - No PPE used by any worker. | - Spirometry: FVC,   FEV₁,  FEV₁/FVC%,  FEF₂₅₋₇₅, PEFR.   - Computerised spirometer (RMS   MED SPIRER  Helios 401); ATS guidelines; best of 3 manoeuvres.   - Workers classified by duration: <5 years (n=56); 5–10 years (n=110); >10 years (n=61). | No | Non-smokers only | - Comparison workers vs   controls  (mean±SD): FVC  (L): 3.02±0.56 vs 3.61±0.46  (p<0.001).   - FEV₁ (L): 2.73±0.50 vs 3.12±0.36 (p<0.001). - FEV₁/FVC (%):   90.40±9.71 vs  84.86±5.71  (p<0.001; HIGHER in  workers — reflects restriction not obstruction).   - FEF₂₅₋₇₅ (L/s):   3.55±1.99 vs 4.0±0.84 (p<0.001).   - PEFR (L/s):   7.28±1.72 vs 8.22±1.1  (p<0.001).   - All three duration groups significantly different from controls for all parameters (p<0.001). - ANOVA by   duration group:  FEV₁/FVC%  declined significantly with duration (F=6.764; p=0.0014).   - FEF₂₅₋₇₅ declined significantly with duration (F=6.274; p=0.0023). - FVC, FEV₁, PEFR not significantly different between duration groups. | • This was the largest pulmonary-function study in this table  (227 workers and 227 matched controls from 53 stations).   - Group findings were consistent with a restrictive pattern. The authors interpreted lower   FEV₁/FVC and  FEF₂₅₋₇₅ after >10 years as a shift toward mixed impairment, but formal clinical classification using an obstruction threshold or lower limit of normal was not reported.   - The higher mean FEV₁/FVC in workers than controls is consistent with the predominantly restrictive group pattern but does not independently establish a diagnosis. - FEF₂₅₋₇₅ and   FEV₁/FVC differed across duration groups, supporting an association with  employment  duration rather than demonstrating longitudinal individual decline.   - Absence of concurrent BTX measurement precludes exposureresponse characterisation or attribution to a |
| **Ref** | **Author, Year Country** | **Population (M/F)** | **Biological Effect Assessed** | **Concurrent Exposure**  **Measured?** | **Smoking**  **Addressed?** | **Quantitative Results** | **Interpretation** |
|  |  |  |  |  |  |  | specific petrol constituent. |

Studies ordered chronologically within each group. "Concurrent Exposure Measured?" = Yes if any quantitative BTX measurement (air, blood, or urine) was performed in the same subjects; No if biological-effect endpoints were measured without concurrent exposure quantification. "Smoking Addressed?" = how smoking status was managed in the study design. Abbreviations: PSA = petrol station attendant; GSA = gasoline station attendant; PPW = petrol pump worker; PBL = peripheral blood lymphocytes; CA = chromosomal aberration; CTA = chromatid-type aberration; CSA = chromosomal-type aberration; MN = micronucleus; CBMN = cytokinesis-block micronucleus; SCE = sister chromatid exchange; CCPI = cell cycle proliferative index; AGT = average generation time; PDT = population doubling time; BN = binucleated cells; BMCyt = buccal micronucleus cytome assay; WCP FISH = whole chromosome painting FISH; OTM = Olive tail moment; TM = tail moment; TI = tail intensity; TL = tail length; FI = fold-increase; HFC = high-frequency cells; 8-oxodG = 8-oxo-7,8-dihydro-2'-deoxyguanosine; AML = acute myeloid leukaemia; MDS = myelodysplastic syndrome; LH = lymphohaematopoietic; SIR = standardised incidence ratio; OR = odds ratio; CI = confidence interval; MetHb = methaemoglobin; PCV = packed cell volume; Hb = haemoglobin; HCT = haematocrit; RBC = red blood cell; WBC = white blood cell; ALT = alanine aminotransferase; AST = aspartate aminotransferase; ALP = alkaline phosphatase; GOT = glutamic-oxaloacetic transaminase; GPT = glutamic-pyruvic transaminase; SOD = superoxide dismutase; GSH = reduced glutathione; MDA = malondialdehyde; CAT = catalase; Hsp70 = heat shock protein 70; TAC = total antioxidant capacity; FRAP = ferric reducing antioxidant power; NO = nitric oxide; TNF-α = tumour necrosis factor alpha; AMCase = acidic mammalian chitinase; NLR = neutrophil-to-lymphocyte ratio; PLR = platelet-to-lymphocyte ratio; IL-8 = interleukin-8; ALA-D = delta-aminolevulinate dehydratase; CD80/CD86 = monocyte activation markers; SPMA = S-phenylmercapturic acid; ttMA = trans,trans-muconic acid; FVC = forced vital capacity; FEV₁ = forced expiratory volume in 1 second; FEF₂₅₋₇₅ = forced expiratory flow rate 25–75% of vital capacity; PEFR = peak expiratory flow rate; PIFR = peak inspiratory flow rate; FIF₅₀ = forced inspiratory flow at 50% vital capacity; ERV = expiratory reserve volume; M = male; F = female; GC-FID = gas chromatography–flame ionisation detection; GC-MS = gas chromatography–mass spectrometry; PID = photoionisation detector; BPDE = benzo[a]pyrene diol epoxide; NLR and PLR reduced in exposed = suggests redistribution of immune cell populations consistent with chronic inflammatory state. *Jakobsson et al. 1993 and Rushton & Romaniuk 1997: values from published abstracts only.
